# Challenges and Prospects of Personalized Healthcare Based on Surface-Enhanced Raman Spectroscopy

**DOI:** 10.34133/research.0572

**Published:** 2024-12-27

**Authors:** Guoqun Li, Xingce Fan, Xiao Tang, Xing Zhao, Qi Hao, Jiaqi Li, Teng Qiu

**Affiliations:** Key Laboratory of Quantum Materials and Devices of Ministry of Education, School of Physics, Southeast University, Nanjing 211189, China.

## Abstract

Personalized healthcare monitoring is a transformative tool for preventing potential risks and enhancing health status, particularly through molecular-level insights. Advances in nanotechnology, smart devices, and artificial intelligence (AI) have revolutionized personalized healthcare, especially in point-of-care testing (POCT), enabling early detection and timely intervention. Recently, surface-enhanced Raman spectroscopy (SERS) technology, particularly with flexible chips, has shown immense promise in this field due to its in situ, rapid, specific, and efficient detection capabilities. In this review, we highlight recent advancements in flexible SERS chips for personalized healthcare monitoring, demonstrating their effectiveness in target sampling and detection. Importantly, we provide a comprehensive overview of potential applications of flexible SERS chips in personalized healthcare, address current challenges, and propose future development directions. We also explore the future development of miniaturized Raman devices to broaden their applications in personalized healthcare monitoring. Additionally, we underscore the important role of AI in enhancing data processing and analysis. Our aim is to offer a thorough guide on integrating SERS into personalized healthcare monitoring, promising a new era of health management.

## Introduction

With economic development and improved quality of life, personalized healthcare monitoring has gained significant attention, essential for addressing individual needs and enhancing health outcomes. Advances in nanotechnology, smart devices, and artificial intelligence (AI) have propelled the evolution of personalized healthcare in point-of-care testing (POCT), enabling daily health monitoring [1–3]. Continuous monitoring facilitates early detection of health changes, allowing timely interventions and preventive measures, particularly for conditions with subtle symptoms that may otherwise go unnoticed [4–6].

Current commercial devices for personalized healthcare monitoring primarily focus on biophysical signals like heart rate and blood pressure. However, compared to physiological information, molecular-level information offers a deeper understanding of health [7,8]. Biosensing techniques, such as electrochemical, colorimetric, and fluorescence methods [9–11], have been applied for specific sensing and diagnostics of biomarkers, but they show some drawbacks, including low sensitivity, susceptibility to interference from complex biological matrices, and the need for specific labels to identify target molecules, impeding their extensive applications. Hence, it is imperative to identify an effective method that comprehensively detects biomolecules and enables integration into personalized healthcare devices.

Surface-enhanced Raman spectroscopy (SERS) has emerged as a promising technology due to its ultrahigh sensitivity and ability to identify molecules based on their intrinsic fingerprint spectra [12–14]. The exploitation of localized surface plasmon resonance (LSPR) of plasmonic nanostructures enables the dramatical Raman enhancement of molecules, which can achieve ultrahigh SERS sensitivity down to single-molecule level, which is particularly favorable for biological detection due to the extremely low concentration of biomarkers [15,16]. Most different from the other sensing technologies, SERS can simultaneously analyze multiple components in a single spectrum with label-free method, providing a comprehensive view of an individual’s health [17]. By using specific labels, SERS can also identify a single specific target, focusing on particular issues [18]. This dual functionality enhances its versatility for both broad health monitoring and specific diagnostics.

However, personalized healthcare monitoring necessitates not only high sensitivity but also the rapid processing of complex samples to adapt to various applications. Biological media like blood, urine, and sweat contain diverse interferents, such as proteins, ions, and various other molecules, that can alter the properties of plasmonic nanoparticles, complicating detection and affecting signal stability and reproducibility [19,20]. Common SERS chips, including colloidal nanoparticles or nanoarrays fabricated on rigid substrates, while effective for biomarker detection [21], often require complex pretreatments to extract target molecules, which may affect the original properties of the target and complicate detection processes [22]. Moreover, other critical aspect of personalized healthcare monitoring is to achieve in situ and rapid POCT detection; traditional rigid SERS chips lack the flexibility to conform to body surfaces, limiting their real-world applications [23]. The challenges of sample handling and adaptation to the human body severely limit the application of SERS technology in personalized healthcare monitoring.

Recently, flexible SERS chips have been developed as a new class of SERS tools, which feature flexibility, porosity, and transparency, making them highly effective in addressing the challenges encountered when applying SERS technology in POCT scenarios [24,25]. These unique properties simplify sample handling and detection, particularly in complex biological conditions, making the process more efficient. Furthermore, their adaptability and portability allow for close contact with irregular biological surfaces, facilitating integration with wearable devices for real-time monitoring, thus promoting their use in next-generation health technologies.

**Fig. 1.**
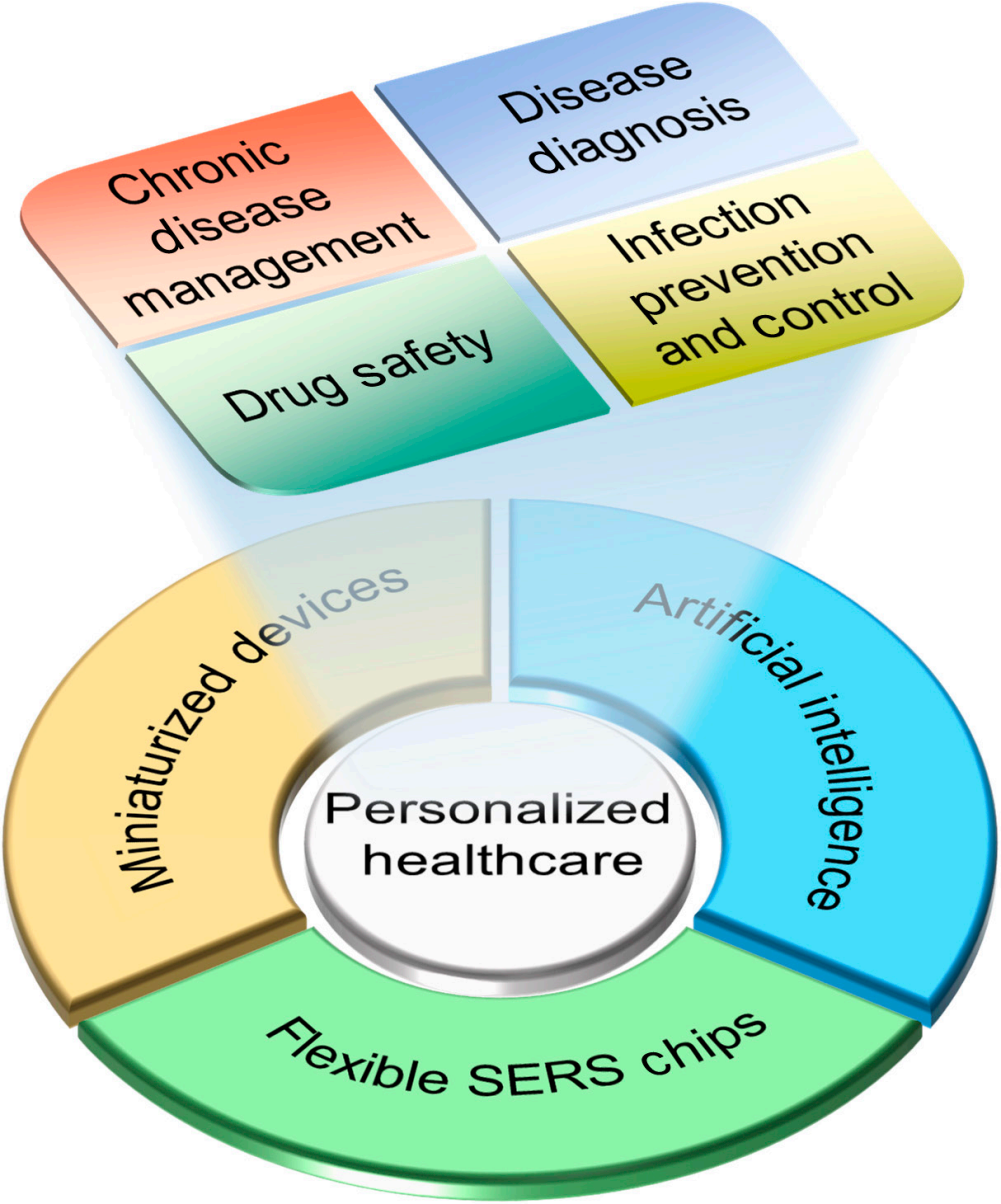
Schematic illustration of SERS-based personalized healthcare monitoring.

In this review, we aim to contribute to the ongoing discourse on integrating SERS into personalized healthcare monitoring, thereby enhancing its application and efficacy. The schematic diagram shown in Fig. 1 provides the overview of this review. First, we provide an in-depth analysis of the development of flexible SERS chips for active target sampling and detection, highlighting key fabrication methods and their importance in advancing SERS technology for personalized healthcare applications. Next, we discuss the broad spectrum of applications for personalized healthcare monitoring and envision the potential role of SERS in this domain. Following this, we highlight the future prospects for SERS in personalized health monitoring, emphasizing the pathway to integrate miniaturized Raman spectrometers for transitioning from laboratory capabilities to home-based healthcare applications. We also underscore advancements in AI for processing and analyzing data, and its critical impact on personalized healthcare monitoring. Our ultimate objective is to promote the broad application of SERS technology in personalized healthcare monitoring.

## Fabricating Flexible SERS Chips: Effective Target Sampling and Detection

The flexible SERS chip retains the key advantages of traditional SERS substrates while expanding its application potential through improved flexibility and adaptability. This increased versatility allows flexible SERS chips to be used in a variety of innovative scenarios, particularly in the fields of personalized healthcare monitoring. In this section, we will discuss several common flexible materials beneficial for personalized healthcare monitoring, including paper, polymers, and metal–organic frameworks (MOFs), which are summarized into Table. They are specially classified into 2 main types of flexible SERS chips: (a) ex vivo SERS chips, designed for sample collection, processing, and detection outside the body, and (b) wearable in situ SERS chips, intended for real-time sampling and continuous monitoring directly from the body.

**Table.** Flexible SERS chips for personalized healthcare monitoring

Types	Structures	Substrate materials	Advantages	Disadvantages
Ex vivo SERS chip	Paper-based testing strips	Paper [29–33]	Cost-effectiveness, portability, biocompatibility, ease of integration, environmental-friendly	Limited durability and stability, easily interfered by environment
	Porous testing chips	Hydrogel [35,36] MOF [37,38]	Variable aperture sizes for molecule screening, high surface area for molecule capture	Limited reproducibility of production, complex fabrication process
Wearable in situ SERS monitoring chips	Polymer-based membranes	PDMS [41] Fibroin [42] Hydrogel [43,44]	High flexibility and conformability to body, easy integration, transparent for in situ monitoring	Bio-fluid aggregation, limited air permeability
	Fiber-based chips	Silk [45] Polyurethane [47] PVA [48]	Lightweight, comfortable, and unobtrusive for long-term wear, high surface area of fiber structure, stretchability	Potential fragility of fibers, complex synthesis processes, batch variability
	Microfluidic devices	PDMS [51,52] Paper [53,54]	Small volumes of bio-fluids, real-time, continuous monitoring, easy-integration into wearable systems	Complexity in device fabrication, potential clogging or leakage in microfluidic channels,
	Microneedle patches	PDMS [57] PMMA [58,59] Hydrogel [60]	Minimally invasive, direct access to interstitial fluid, easy integration with wearable devices	Potential for skin irritation, complex fabrication process

### Ex vivo SERS chip: Sample processing and detection

Ex vivo SERS detection involves pretreatment of biological samples, such as blood, urine, or sweat from the body, and detecting them externally using SERS technology [21]. This method creates a controlled environment that enhances the Raman signals of target molecules, allowing for highly sensitive and selective detection. When combined with flexible materials that possess unique properties like hydrophilicity and porosity, the sample pretreatment can be simplified and the SERS detection performances would also be significantly promoted, thereby ensuring more accurate and reliable measurements for personalized healthcare monitoring.

#### Ex vivo paper-based SERS testing strips

Paper is an affordable and highly flexible material, making it ideal for SERS applications. Sourced from natural materials, these sensors are biodegradable and nontoxic, promoting environmental sustainability and user safety [26]. The natural porosity and hydrophilicity of paper enhance analyte retention, increasing interaction with nanostructures and improving SERS sensitivity [27]. Additionally, the customizable nature of paper allows for integration into various formats, enhancing the versatility of paper-based SERS chips. For instance, paper-based lateral flow immunoassay (LFIA) strips are well-known ex vivo diagnostic tools valued for their simplicity and rapid results [28]. Engineered to detect target analytes within minutes, these platforms are highly suitable for POCT. When integrated with SERS, LFIA strips achieve enhanced sensitivity and specificity, enabling precise detection of smaller analyte quantities [29,30].

**Fig. 2.**
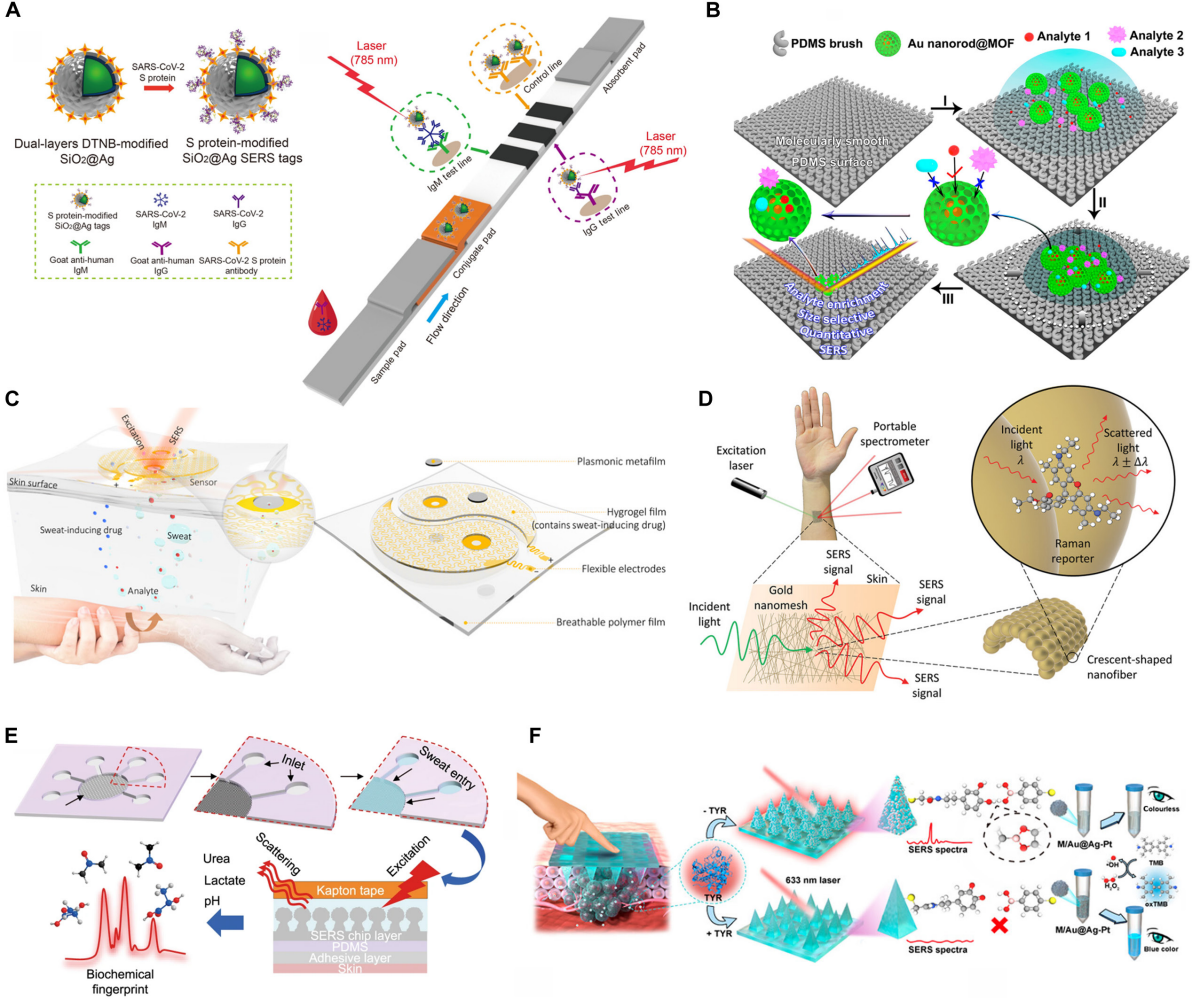
Flexible SERS chips for target sampling and detection. (A) Schematic illustration of paper-based SERS diagnostic strips utilizing LFIA. Reproduced with permission from [31]. Copyright 2021, Elsevier. (B) Representation of porous MOF structures encapsulating Au nanorods with slippery surface for efficient macromolecule exclusion and selective small-molecule detection in complex environment. Reproduced with permission from [37]. Copyright 2020, American Chemical Society. (C) Sketch of wearable flexible polymer SERS membranes integrated with plasmonic metafilms to monitor metabolites in sweat. Reproduced with permission from [41]. Copyright 2020, Science. (D) Diagram of wearable polymer nanofibers fabricated via electrospinning. Reproduced with permission from [48]. Copyright 2022, Wiley. (E) Schematic of wearable microfluidic devices made from flexible polymers for sweat analysis. Reproduced with permission from [51]. Copyright 2022, Nature. (F) Illustration of wearable SERS microneedle (MN) patches for effective sampling interstitial fluid and detection. Reproduced with permission from [57]. Copyright 2023, American Chemical Society.

As depicted in Fig. 2A, SERS-based LFIA strips have been developed for the application of coronavirus (COVID-19), realizing simultaneous detection of anti-SARS-CoV-2 immunoglobulin M (IgM) and IgG antibodies. This was achieved using advanced SERS tags, consisting of SiO_2_ cores coated with Ag shells (SiO_2_@Ag) and labeled with dual layers of Raman dye. These tags were conjugated with anti-human IgM/IgG antibodies and immobilized onto separate test lines of the strip. The inherent rapid fluid transport capabilities of the paper-based platform significantly reduce the required sample volume, thereby facilitating efficient target–receptor interactions. The platform demonstrated exceptional sensitivity, achieving a detection limit 800 times lower than conventional LFIA strips employing gold nanoparticles. Clinical validation using serum samples from 19 COVID-19-positive and 49 healthy individuals confirmed the platform’s high accuracy and specificity, underscoring its potential as a reliable tool for clinical diagnostics [31].

These strips leverage the high specificity of receptor–ligand interactions, making them broadly applicable across various diagnostic settings. Their compatibility with portable devices enhances their practicality. For example, when combined with a portable SERS-based LFIA reader featuring multiplexed detection through an integrated reaction column, this system can simultaneously detect multiple biomarkers or analyze several samples in parallel. Utilizing this design, the reader effectively detected specific biomarkers, such as α-fetoprotein, carcinoembryonic antigen, and prostate-specific antigen (PSA), demonstrating its effectiveness in multi-target detection and potential for streamlined, high-sensitivity diagnostics [32].

Furthermore, paper-based SERS strips are well suited for cost-effective mass production through techniques such as evaporation deposition, coating, or printing. The naturally rough surface of paper enhances the formation of densely packed hotspots, further improving the sensitivity of molecular detection. A notable example is a paper-based platform developed by printing technology for low-cost, rapid on-site detection of illicit drugs in urine. In this system, filter paper was coated with chitosan to enhance surface smoothness and improve Ag ink adhesion. The substrate was modified via binary silylation to boost sensitivity by concentrating analytes. SERS-active Ag ink can achieve a detection limit of 1.43 parts per billion (ppb) for methylamphetamine using a portable Raman spectrometer, with a strong correlation index (*R*^2^ = 0.9927) in urine samples [33].

Paper-based SERS strips hold great promise for next-generation personalized healthcare monitoring, offering cost-effective, portable, and highly sensitive diagnostic solutions. Especially, roll-to-roll manufacturing is anticipated to become a feasible approach, enabling scalable and efficient production of paper-based SERS strips. However, challenges still remain in ensuring the durability, stability, and long-term performance of these strips. Particularly, environmental factors, like humidity, temperature, and handling, can degrade the plasmonic structures, compromising sensitivity and reproducibility. To address this, protective coatings, chemical modifications, or hydrophobic treatments could enhance the strips’ stability and extend their operational lifespan. Furthermore, ensuring consistent hotspot generation and uniformity across batches is crucial for reliable performance. Techniques such as transferring or templating can improve the stability and diversity of plasmonic structures, further enhancing their performance. Addressing these challenges through innovative material design and process optimization will be essential to fully realize the potential of paper-based SERS strips in personalized healthcare monitoring applications.

#### Ex vivo porous SERS testing chips

Embedding plasmonic nanoparticles into porous flexible materials, such as hydrogels and MOFs, significantly enhances the stability of nanoparticles in SERS chips [34]. This approach also facilitates selective screening by utilizing variable aperture sizes, streamlining the pretreatment process. Additionally, it offers a large surface area with abundant adsorption sites, further enhancing SERS performance. For instance, hydrogels can be coated on plasmonic nanoparticles, with nanopore sizes controlled between 4.6 and 6.6 nm through crosslinking to screen out larger molecules like proteins (7.1 nm), thus avoiding complex pretreatment. The plasmonic nanoparticles are embedded in a 3-dimensional (3D) hydrogel framework, effectively capturing targets within plasmonic nanogaps. The combination of size selectivity and molecular enrichment in hotspots allows for direct and highly sensitive detection of pyocyanin in aqueous solutions of bovine serum albumin and human serum [35].

Aperture screening not only is effective for detecting small molecules but also provides an ideal strategy for larger biomolecules. The detection of proteins, often limited by their size, restricts access to the narrow spaces of SERS hotspots. To address this challenge, Au nanotriangle plate arrays are embedded in a thermoresponsive hydrogel to create a flexible SERS chip for protein sensing. This innovative design employs a gel filter trapping strategy that utilizes the unique properties of hydrogels, including their water-absorbing capabilities and 3D polymer networks, to effectively separate biopolymers. This approach facilitates the movement of proteins into hotspot regions, achieving ultrahigh SERS sensitivity for protein detection and enabling identification at the single-molecule level [36].

Beyond hydrogel SERS chips, emerging porous materials like MOFs can be engineered as secondary structures for plasmonic nanoparticles or nanoarrays, enhancing nanoparticle stability and enabling a screening effect. As shown in Fig. 2B, encapsulating thick MOF shells around Au nanorods effectively mitigates uneven particle aggregation, improving quantification capabilities, though at the expense of sensitivity. The introduction of a slippery surface compensates for this sensitivity reduction through an analyte enrichment mechanism. The porous structure of the MOF shell selectively permits only analytes smaller than its pore size to access the nanorods, contributing to the SERS signal in complex sample matrices. This integrated SERS platform merges analyte enrichment and filtration functions, facilitating sensitive, quantitative, and size-selective identification of analytes in intricate environments. Notably, it enables the selective detection of 4-nitrobenzenethiol at nanomolar concentrations in whole blood, demonstrating substantial potential for disease diagnostics and allowing for the analysis of biomarkers in biofluids by modulating the aperture size of MOF shell [37].

Furthermore, the expansive surface area of porous structures enhances analyte adsorption. For instance, core-shell Au@MOF nanostructures have been developed for detecting gaseous benzaldehyde in breath samples by combining mesoporous Au (MesoAu) with zinc imidazole framework-8 (ZIF-8) MOFs. The MesoAu component provides numerous active sites and interconnected mesopores, facilitating the diffusion of analytes for SERS detection. The ZIF-8 shell further concentrates target molecules around the MesoAu, significantly boosting SERS sensitivity. This architecture achieved a detection limit of 0.32 ppb for gaseous benzaldehyde, highlighting its promising potential for the rapid diagnosis of early-stage lung cancer [38].

The development of porous SERS testing chips simplifies sample handling and enhances analyte collection and analysis. Their high surface area and abundant adsorption sites are critical for increased sensitivity and efficient molecular detection. However, the complex fabrication processes pose challenges in achieving consistent batch production with variations in pore size, distribution, and plasmonic structure, which will affect reproducibility of SERS chips. Therefore, achieving precise control over pore size and thickness is essential for addressing these challenges. In particular, the growth of porous structures on the surface of individual particles offers a promising approach to increasing the diversity of plasmonic structures. This strategy is expected to improve the stability and consistency of the detection process, ensuring more reliable results. Ongoing optimization of fabrication techniques is key to streamlining production, improving yield, and ensuring consistent high-quality results, which facilitate broader use of porous SERS chips in diagnostics.

### Wearable in situ SERS monitoring chips: Real-time sampling and monitoring

Wearable in situ monitoring offers a significant advantage in ensuring the timeliness and accuracy of detection, as it eliminates the potential damage and contamination associated with sample extraction. Traditional SERS chips with rigid substrates are unable to conform to the body’s irregular shapes, which limits their effectiveness for in situ sampling and detection. In contrast, flexible materials that are soft, lightweight, and transparent provide better contact with body surfaces, making them ideal for wearable monitoring. This section reviews and discusses various typical wearable in situ SERS chips, including polymer-based membranes, fiber-based chips, microfluidic devices, and microneedle patches.

#### Wearable polymer-based SERS membranes

Wearable flexible polymer SERS membranes that combine plasmonic nanostructures with transparent polymer materials, such as polydimethylsiloxane (PDMS), polymethyl methacrylate (PMMA), or various hydrogels, provide a straightforward solution for in situ, noninvasive personalized healthcare monitoring [39]. These SERS chips can be easily fabricated using techniques like spin-coating or dip-coating [40]. As shown in Fig. 2C, the chip incorporates an ordered array of Ag nanocubes within the PDMS membrane, functioning as the SERS sensing element. These membrane SERS chips can adhere to body tissues and retain plasmonic activity despite various deformations, enabling effective in situ SERS detection of biomarkers in sweat [41].

Various templating and cutting techniques can tailor polymer membranes for specific applications, such as contact lenses designed for in situ tear collection and detection. A novel SERS contact lens material has been developed for selective glucose detection in human tears. This SERS lens material features a layered structure that includes a silk fibroin layer, Ag nanowires coated with 4-mercaptophenyl boronic acid (MPBA), and a protective film. The silk fibroin layer acts as a biocompatible interface that filters out larger tear proteins, while the MPBA-coated Ag nanowires create hotspots that enhance Raman signals and selectively bind glucose through cis-diol complexation. The SERS lens material demonstrated excellent glucose sensing capabilities, with a concentration range of 500 nM to 1 mM and a detection limit of 211 nM [42]. However, the polymer membrane usually face limitations, such as localized aggregation of biological fluids, restricted air permeability, and limited flexibility.

In recent years, hydrogel materials have emerged as an effective choice for wearable chips due to their excellent hydrophilicity and biocompatibility [6]. Their hydrophilic nature enables the rapid transport of biological fluids, preventing accumulation and ensuring efficient sample handling. Furthermore, hydrogels provide effective storage for biological fluids, minimizing sample loss and reducing detection time, making them highly suitable for real-time and accurate wearable monitoring applications. A notable example is a wearable SERS chip with a hydrogel membrane incorporating plasmonic trimers for noninvasive uric acid monitoring in sweat. These plasmonic trimers, with nanogaps under 5 nm, generate strong LSPR that significantly amplifies Raman signals. The hydrophilic hydrogel membrane pumps sweat through the nanogaps, which increase the capturing of molecules and decrease the detection time within 5 min. The synergistic effect between trimers and hydrogel improves the sensitivity of biomarkers in sweat, which realize the effective detection of uric acid in sweat [43]. Another example is a hydrogel-based SERS sensor in nappies for rapid urine biomarker detection, targeting creatinine, uric acid, bilirubin, and pH. The hydrogel retains water for sample freshness, while the embedded Ag nanostructures enhance SERS sensitivity by creating plasmonic hotspots and offering antimicrobial properties. The SERS sensor achieved a detection limit of 0.59 μM for creatinine, 69 nM for uric acid, and 89 nM for bilirubin, demonstrating its effectiveness in wearable SERS applications [44].

In summary, wearable flexible polymer-based SERS membranes represent a foundational approach to achieving personalized healthcare monitoring in POCT. By integrating polymers with plasmonic nanostructures, they enhance functionality and safeguard nanostructures. Hydrogels, known for their hydrophilicity, facilitate rapid fluid transport and storage, boosting detection efficiency and sensitivity. However, challenges such as uneven plasmonic nanostructure distribution, instability under physiological conditions, fluid aggregation, limited air permeability, and inadequate mechanical robustness hinder their broader application. Addressing these challenges will be critical for advancing wearable SERS membranes, enabling widespread use in POCT, and improving diagnostic accuracy and accessibility. Therefore, we offer some possible solutions. Hard mask-assisted assembly of noble metal nanostructures may also supply a good solution to the uneven distribution of plasmonic nanostructures, promoting the SERS signal uniformity and reproducibility. Advanced fabrication techniques, like electrospinning, 3D printing, and layer-by-layer assembly, offer more robust and functional design concept to strengthen the stability and minimize the fluid aggregation. Besides, incorporating stimuli-responsive materials may further enable dynamic sensing for varied physiological conditions.

#### Wearable fiber-based SERS chips

Fiber-based SERS chips offer significant advantages for wearable personalized health monitoring, where the flexible structure conforms well to the skin, enhancing comfort and enabling continuous monitoring of biomarkers in body fluids for real-time health assessment. For example, a versatile wearable sensor based on functionalized silk fiber has been developed with dual capabilities for biomechanical and biomolecular sensing. By incorporating Ag nanoparticles, this sensor achieves both a piezoresistive response and LSPR, enabling precise detection of mechanical pressure and specific biomolecular targets. Such multimodal functionality supports comprehensive health monitoring, capable of identifying muscle strain and potentially extending its applications to the diagnosis and monitoring of neuromuscular disorders like amyotrophic lateral sclerosis [45].

Especially, the fiber-based SERS chips exhibit better performances due to high surface area-to-volume ratio, significantly enhancing target molecule interaction and improving detection sensitivity. The typical fabricating method of nanofibers is electrospinning, where a polymer solution is ejected through a syringe needle under a high-voltage electric field, producing ultra-fine nanofibers with high surface area-to-volume ratio and providing more adsorb sites for molecules [46]. The plasmonic nanoparticles can be premixed into the polymer precursor solution or deposited on the surface after the nanofibers are made. For example, a flexible and nanofibrous wearable SERS chip was fabricated using electrospinning thermoplastic polyurethane with an Au sputter coating, enabling sweat pH monitoring. Functionalized with 4-mercaptobenzoic acid (4-MBA) and 4-mercaptopyridine (4-MPy), it achieved high sensitivity with resolutions of 0.14 and 0.51 pH units, respectively, by using just 1 μl of sweat. The chip demonstrates stable performance over 35 d and exhibits rapid sweat absorption, repeatability, and reversibility, making it highly effective for wearable health monitoring [47].

Additionally, the polymer fiber-based SERS chips possess inherent stretchability, making it ideal for integration into wearable devices, ensuring consistent and reliable detection during real-time health monitoring. For instance, a highly scalable, wearable SERS sensor was developed using a flexible, stretchable, adhesive, and biointegratable Au fiber network. This sensor was created by depositing Au onto polyvinyl alcohol (PVA) polymer nanofibers, forming a robust and flexible structure, as shown in Fig. 2D. This SERS chip can be fabricated into any shape and adhered to virtually any surface, making it suitable for label-free, large-scale, in situ detection of various analytes across a wide concentration range [48].

In summary, fiber-based SERS chips offer key advantages, including flexibility, high surface area, and stretchability, which enhance electromagnetic fields and provide ample molecular adsorption sites. Their fibrous structure enables seamless integration into textiles for noninvasive, real-time biomarker detection, making them ideal for wearable healthcare monitoring. Despite these benefits, challenges such as complex synthesis processes, batch variability, stability, and inconsistent fiber alignment limit their reliability and scalability. To address these issues, advanced fabrication methods like electrospinning and 3D weaving have been developed to improve uniformity and reproducibility. Protective coatings and embedding plasmonic nanostructures within fibers enhance stability and durability. Overcoming these hurdles is essential to unlock the full potential of fiber-based SERS chips, paving the way for their broader application in personalized healthcare.

#### Wearable microfluidic SERS devices

Beyond material and structural challenges, the low concentrations and small volumes of biological fluids pose significant difficulties for wearable SERS chip applications. Wearable microfluidic SERS devices offer a promising solution by employing narrow channels, typically ranging from tens to hundreds of micrometers, to facilitate precise fluid manipulation at the microscale. These devices integrate multiple functions, including mixing, separation, and detection, on a single platform, allowing for real-time analysis of biomarkers [49].

Microfluidics enhances sensitivity and specificity by optimizing surface area-to-volume ratios, facilitating efficient interactions between samples and SERS substrates. Flexible polymers like PDMS are commonly used to fabricate channels through methods, such as soft lithography, photolithography, 3D printing, or templating [50]. Noble metal nanoparticles are then deposited within these channels to create functional nanoarrays. For example, as shown in Fig. 2E, these nanoarrays enable controlled sweat sampling and real-time analysis of biomarkers like urea, lactate, and pH through continuous sample refreshment. The inherent plasticity of the polymers allows for precise construction of microfluidic channels, enabling controllable sweat administration with high temporal resolution and facilitating ongoing analysis of sweat biomarkers [51]. Moreover, incorporating multiple channels enhances the device’s capabilities by enabling simultaneous detection of different biomarkers, such as sweat volume, pH, and lactate levels [52].

In addition to polymer materials, paper-based microfluidics offers a versatile alternative, easily fabricated by cutting paper into microfluidic shapes. The inherent hydrophilicity of paper promotes fluid movement through the channels, allowing for the integration of SERS sensors to detect specific biomarkers. This approach is widely applied in wearable sweat detection, enabling analysis of sweat rate and identification of markers like uric acid [53]. Paper-based microfluidic devices utilize the material’s natural hydrophilicity to capture and drive sweat, facilitating detection and quantification of various biomarkers [54,55].

In summary, microfluidic SERS devices offer transformative potential for wearable personalized healthcare monitoring by enabling real-time biomarker analysis with minimal sample volumes. Their compact design facilitates seamless integration into wearable systems, supporting continuous health monitoring. The controlled fluid dynamics within microchannels enhance analyte transport and Raman signal amplification, improving detection sensitivity and accuracy. Despite these benefits, challenges such as microchannel clogging and design inconsistencies must be addressed. Advanced fabrication techniques, such as 3D printing and laser-assisted manufacturing, can enhance microchannel precision. Hydrophilic or antifouling coatings can prevent clogging, while flexible, durable substrates improve mechanical stability for wearable applications. Integrating smart materials or adaptive feedback systems can further bolster reliability under dynamic conditions. Addressing these challenges will be pivotal in advancing microfluidic SERS devices, enabling broader adoption in personalized healthcare monitoring.

#### Wearable microneedle SERS patches

In recent years, wearable microneedle SERS patches have gained attention for their painless, bloodless, and direct in situ biomarker monitoring in interstitial fluid. Fabricated through techniques like template molding or 3D printing, these microneedle patches possess enough hardness to penetrate the skin [56]. For example, integrating with trimetallic Au@Ag-Pt nanoparticles enable dual-mode SERS and colorimetric detection of tyrosinase, showing promise for melanoma screening. As shown in Fig. 2F, the microneedle patch contacts suspicious skin lesions to monitor real-time tyrosinase activity for early diagnosis. Modified with dopamine, the microneedles interact with the Raman reporter 4-MPBA-labeled Au@Ag-Pt nanozyme. Upon piercing tyrosinase-positive skin, catechol is oxidized to benzoquinone, disrupting the ester bond and deactivating the SERS signal. Simultaneously, unbound Au@Ag-Pt catalyzes the conversion of colorless 3,3′,5,5′-tetramethylbenzidine (TMB) to blue oxidized TMB (oxTMB), producing a weak colorimetric signal. This dual-detection mechanism enhances the accuracy of real-time tyrosinase monitoring for melanoma detection [57].

Additionally, plasmonic nanoparticles can be preintegrated into microneedle arrays for in situ detection, eliminating additional steps like stripping. For example, a low-cost PMMA microneedle array was developed for glucose detection by a simple micro-molding method, featuring good light transmission and mechanical strength. The microneedles were coated with Ag nanoparticles and treated with 1-decanethiol, enabling direct measurement of glucose in skin interstitial fluid within several minutes [58]. A similar strategy was employed to monitor acute peritonitis using a SERS-tagged microneedle patch. This patch, comprising core-satellite Au nanoparticles and 3-mercaptophenylboronic acid Raman reporter, exhibits high SERS sensitivity and selectivity for hydrogen peroxide (H_2_O_2_), a key indicator of peritonitis development. This microneedle patch not only reliably monitors the different stages of peritonitis but also evaluates the efficacy of treatments. The altered SERS signal aligns with changes in plasma pro-inflammatory factors [such as tumor necrosis factor-α (TNF-α)] and peritoneal pathological manifestations, providing a comprehensive assessment of disease progression and therapeutic outcomes [59].

Furthermore, incorporating porous structures or hydrophilic materials, like hydrogels, enhances microneedles by facilitating interstitial fluid extraction. For instance, hollow microneedle patches can draw subcutaneous fluids using negative pressure by a simple finger push. When combined with microfluidic channels, these fluids can be routed to a chamber containing high-density 3D Au nanoarrays for SERS detection, enabling ultrasensitive, label-free detection of uric acid with a limit of 0.51 μM. This system also allows for rapid identification of target molecules when integrated with a portable Raman spectrometer [60].

In summary, microneedle SERS patches offer significant advantages for wearable personalized health monitoring due to their minimally invasive design, enhancing comfort and user adherence. By efficiently collecting biomarkers from interstitial fluid, they enable real-time health assessments and integrate seamlessly into wearable systems. However, challenges include fabrication inconsistencies, such as variability in dimensions and surface coatings, which can affect signal reliability. Additionally, variations in user application, such as differences in pressure during attachment, or individual skin conditions, may influence biomarker extraction efficiency. Furthermore, prolonged wear or repeated use can lead to skin irritation or localized inflammation, posing potential risks for users. To overcome these issues, advances in fabrication techniques, such as lithography, laser micromachining, or 3D printing, have been explored to improve precision and uniformity in microneedle production. Coating microneedles with biocompatible or stimuli-responsive materials such as hydrogel can enhance biomarker capture while minimizing irritation. Addressing these challenges will be crucial to fully realize the potential of microneedle SERS patches in wearable personalized health monitoring.

## Applications of Flexible SERS Chips in Personalized Healthcare Monitoring: Real-World Impacts and Innovations

The goal of personalized healthcare is to provide timely insights into an individual’s health through rapid, continuous, and effective monitoring, enabling early intervention for potential issues. Various biomarkers, such as metabolites, proteins, nucleic acids, drugs, and certain viruses and bacteria, are closely linked to human health. Flexible SERS chips have emerged as exceptional tools for the rapid, sensitive, and selective detection of these biomarkers. Their application in personalized healthcare monitoring has shown significant promise in areas like chronic disease management, disease diagnosis, drug safety monitoring, and infectious disease prevention and control.

### Detection mechanisms

#### Label-free SERS method: Simplicity and broad applicability

Label-free SERS method directly detects the intrinsic Raman signals from the target molecules, broadening its applicability by eliminating the need for complex labeling procedures. This method leverages the characteristic vibrational frequencies of chemical bonds to identify targets, making it suitable for comprehensive analysis of complex biomarkers in biofluids [17]. However, many molecules in biological fluids compete for adsorption sites, which can affect detection accuracy, and proper pretreatment is necessary. For example, detecting drug molecules in sweat, a wearable platform consisting of a plasmonic Ag nanowire layer integrated with a silk fibroin protein film enables efficient screening and detection. The Ag nanowire layer generates robust 3D hotspots, while the fibroin protein film adsorbs aqueous solutions and selectively filters molecules larger than the nanopores in its β-sheet matrix. This configuration effectively captures the unique spectral fingerprint of 2-fluoro-methamphetamine within complex biofluids, enhancing specificity and detection accuracy in real-world applications [61].

Additionally, the development of advanced algorithms will further enhance the sensitivity and specificity of label-free SERS detection. However, this method faces challenges when dealing with molecules that have similar structure and composition, particularly large biomolecules such as proteins and DNA, which can produce indistinguishable spectra and hinder accurate analysis [62]. Molecular adsorption on plasmonic nanoparticles can also influence the signal, especially for low-affinity and large biomolecules. Furthermore, the high complexity of biological samples, containing a wide and dynamic range of biomolecules, may lead to interference and interactions with the SERS chip, impacting both sensitivity and quantification [63].

#### Label-based SERS method: Enhanced sensitivity and specificity

Label-based SERS method is an indirect detection strategy that utilizes Raman reporter molecules to label the SERS tags, enabling reliable quantification and multiplexing of low-abundance analytes. The signal originates from the probe or label, which provides amplified signals and specific binding to the target. This approach is particularly advantageous for detecting biomarkers with similar compositions, and targets with poor cross sections or low affinity [18,64].

For instance, direct detection of glucose through SERS is challenging. By employing boronic acid-based recognition molecules on a functionalized surface, glucose forms boronate esters reversibly and is captured on the surface. In this method, boronic acid molecules act as both glucose recognition structures and Raman-active molecules [42]. Another approach involves enzymatic reactions, where the product, such as hydrogen peroxide, can convert a Raman-caged molecule to a Raman-active product, inducing signal changes for efficient glucose monitoring [65]. Additionally, dye molecular tags can be used in methods such as LFIA strips, where dye molecules modify antibodies or nanoparticles to detect specific RNA or DNA [66]. This targeted approach is tailored to specific needs and focuses on identifying particular targets. However, label-based detection increases the complexity and cost of detection to some extent. The probes used for labeling can be susceptible to contamination or interference, potentially affecting the detection performance.

### Chronic disease management: Continuous and noninvasive monitoring

Chronic diseases, like gout, diabetes, and cardiovascular conditions, pose significant health challenges that necessitate regular and precise monitoring for effective management and progression mitigation. These conditions are often linked to key metabolites such as uric acid, glucose, and cholesterol. Using these typical chronic diseases as examples, we will make discussions on the application of flexible SERS chips in personalized health monitoring.

**Fig. 3.**
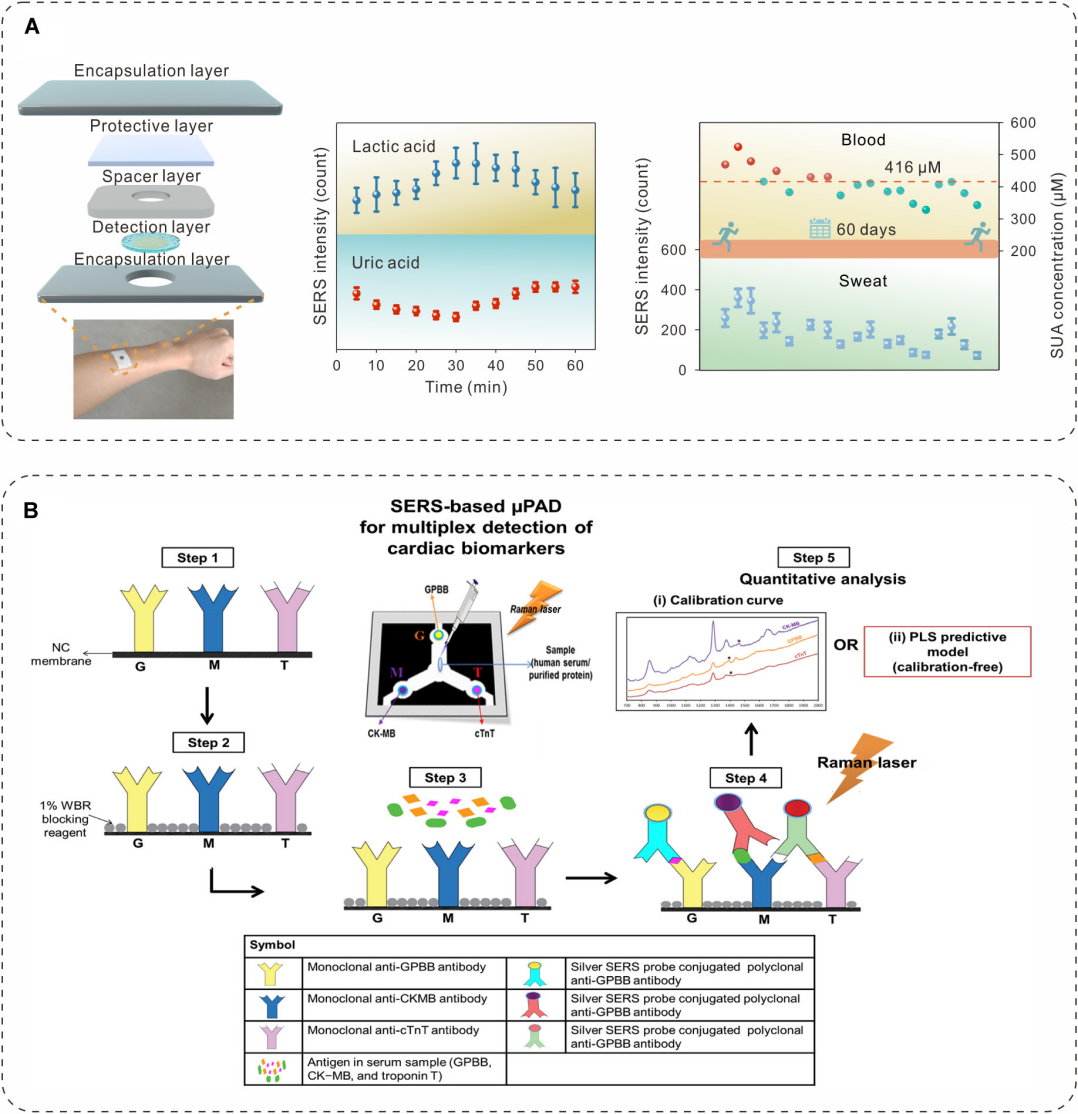
(A) Schematic representation of continuous uric acid monitoring for analysis of the relationship with exercise using a wearable hydrogel SERS chip. Reproduced with permission from [43]. Copyright 2024, American Chemical Society. (B) Schematic illustration of cardiac biomarker detection for disease diagnosis at various testing sites using a SERS-based microfluidic paper analytical device (μPAD). Reproduced with permission from [73]. Copyright 2020, Elsevier.

Gout management relies on continuous monitoring of uric acid levels, which is crucial for preventing flare-ups. Uric acid is the typical marker that usually can be detected in label-free method. For example, the uric acid was detected by a wearable paper-based microfluidic chips, enabling dynamic testing at multiple sites, providing real-time insights into uric acid metabolism for timely interventions. This approach enhances uric acid regulation and helps prevent symptom recurrence [53]. To improve uric acid management, our team specifically investigated the impact of exercise on uric acid levels in the body using a wearable hydrogel SERS chip. Importantly, based on label-free method, we carried out a comprehensive analysis of the sweat spectrum, a more comprehensive analysis of the mutual interference between metabolites. As illustrated in Fig. 3A, we found that high-intensity anaerobic exercise may temporarily suppress uric acid metabolism due to competition with lactic acid. However, long-term regular exercise effectively reduces serum uric acid levels, providing valuable guidance for uric acid management. Notably, we also established a correlation between uric acid levels in sweat and serum, confirming the effectiveness of sweat analysis for monitoring uric acid [43]. Establishing robust links between biomarkers in blood and other biological fluids, along with their relationships to physiological processes, is essential for advancing SERS applications in personalized health monitoring. Additionally, the integration of AI technology with flexible SERS chips has enhanced the detection and management of gout. For example, SERS spectra of uric acid in human sweat were collected using a wearable smart platform and analyzed with AI algorithms, achieving an impressive identification accuracy of 97%. This innovative approach significantly improves the precision and efficiency of gout detection, paving the way for early intervention and personalized treatment strategies [67].

Diabetes, another leading chronic condition, demands precise blood glucose monitoring for effective management. Given the complex composition of blood, developing a simple but effective method for blood glucose detection is highly desirable. For instance, a SERS-hydrogel micro-pellet enables glucose detection at concentrations as low as 10 μM in whole blood. These micro-pellets were designed with selective pore sizes that allow the passage of small molecules while excluding larger ones, which enhanced the selectivity of SERS for small-molecule detection. Utilizing the silver mirror reaction, glucose reduces Ag^+^ ions to form silver deposits, which amplify the signal of the probe molecule 4-mercaptobenzonitrile, thereby enabling accurate and efficient glucose concentration measurement [68]. Considering the inconvenience of blood testing, alternative methods such as glucose monitoring through tears, urine, sweat, or interstitial fluid have gained attention due to their correlation with blood glucose levels. For instance, a hydrogel-based SERS chip has successfully detected glucose in sweat within a range of 0.01 to 5 mM, with a high linear correlation (*R*^2^ = 0.9923). This chip, featuring gap-enhanced Au nanopetals functionalized with 3-mercaptopropylboronic acid, utilizes an enzyme-catalyzed glucose reaction that produces H_2_O_2_. This H_2_O_2_ then converts phenylboronic acid to phenol, enabling sensitive and reliable glucose analysis [69].

Cardiovascular diseases are often linked to abnormal cholesterol levels, with high cholesterol contributing to hypertension, myocardial infarction, coronary heart disease, and atherosclerosis, while low levels are associated with hyperthyroidism, anemia, and cancer. For instance, cholesterol levels in human serum can be accurately monitored using a nanomaterial-based artificial enzyme system with a dynamic detection range of 1 to 100 μM and a detection limit of 0.36 μM. This system utilizes Ag nanoparticles synthesized on the surface of the MIL-101(Fe) MOF, which facilitates the enzyme-catalyzed reaction producing H_2_O_2_. In the presence of target cholesterol, cholesterol oxidase catalyzes the oxidation of cholesterol, generating H_2_O_2_. Concurrently, MIL-101(Fe) catalyzes this H_2_O_2_ to oxidize the initially non-Raman-active leucomalachite green into its Raman-active form, malachite green, enabling precise cholesterol detection through SERS [70].

### Disease diagnosis: Early detection and intervention

Certain underlying diseases, such as cancer and neurological or genetic disorders, necessitate rapid and effective diagnostic methods [71]. Diagnoses often rely on specific biomarkers; for instance, various cancers are identified by detecting unique proteins, DNA, or RNA. The high sensitivity of SERS allows for the detection of disease biomarkers at extremely low concentrations, essential for early diagnosis. Functionalizing plasmonic nanoparticle surface to selectively bind disease biomarkers enhances SERS accuracy by improving specificity and reducing false positives. For example, a plasmonic trimer chip with high sensitivity and a trap effect has been developed for distinguishing adenocarcinoma, squamous carcinoma, and benign cases. This chip leverages the specific reaction between aldehydes and 4-aminothiophenol to enable sensitive detection of aldehyde biomarkers. By analyzing the concentration of aldehydes associated with different lung cancer types, this platform can accurately identify and classify adenocarcinoma, squamous carcinoma, and benign cases, supporting precise diagnostic differentiation [72].

To enhance diagnostic accuracy, the simultaneous detection of multiple biomarkers offers a significant advantage. For example, in diagnosing acute myocardial infarction (AMI), typical cardiac biomarkers like glycogen phosphorylase isoenzyme BB (GPBB), troponin T (cTnT), and creatine kinase-MB (CK-MB) require precise and rapid detection. As illustrated in Fig. 3B, microfluidic paper-based devices (μPADs) embedded with specific receptors for each biomarker provide selective detection. Using distinct Raman reporters (4-nitroaniline, tert-butylhydroquinone, and methyl red), the μPADs facilitate multiplexed quantification of these biomarkers. Integrating partial least squares (PLS) predictive models allows for accurate quantification of low biomarker concentrations (8, 10, and 1 pg/ml for GPBB, CK-MB, and cTnT, respectively), which supports early AMI diagnosis and demonstrates the platform’s potential for sensitive, quantitative biomarker detection [73].

Additionally, combining flexible SERS chips with AI enables a straightforward, label-free approach for disease diagnosis. For instance, a paper-based SERS chip featuring a 3D plasmonic coral nanoarchitecture has been developed to directly classify urine samples from prostate and pancreatic cancer patients. This chip facilitates the identification of abnormal samples with high sensitivity and specificity. The SERS spectra are analyzed using supervised classification through deep learning models, including recurrent neural networks (RNNs) and convolutional neural networks (CNNs), which successfully distinguish between prostate and pancreatic cancer patients and healthy individuals. This approach shows promise for POCT when integrated with a handheld Raman spectrometer, offering a portable and efficient diagnostic solution [74].

### Drug safety: Monitoring and optimization

Detecting and managing drug levels is crucial for effective disease treatment [75], especially for high-risk medications like fentanyl, where overdose can cause severe consequences. SERS-based analysis in human biofluids faces challenges from fouling by endogenous biomolecules that adsorb onto substrates. To combat this, a flexible plasmonic patch using poly(ethylene glycol)-thiolate functionalized with Au triangular prisms on adhesive tape was developed. This design enhances sensitivity while resisting fouling, allowing accurate drug detection in human plasma, which enable label-free and direct detection of drug molecules. The chip enabled the detection of potent drugs such as 4-anilino-N-phenethyl-piperidine, cocaine, heroin, and JWH-018, facilitating potent drug detection in human plasma, helping ensure that drug levels remain within the therapeutic range [76].

**Fig. 4.**
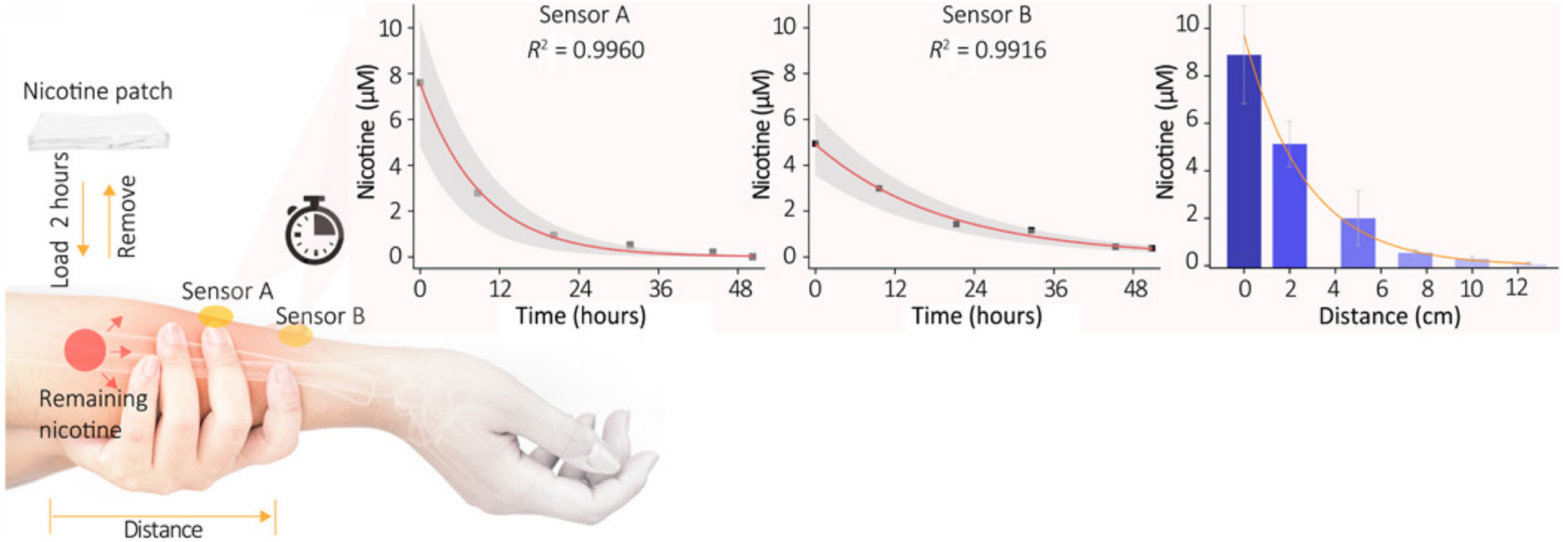
Schematic of drug metabolism monitoring in sweat for drug safety with a wearable polymer membrane chip. Reproduced with permission from [41]. Copyright 2021, Science.

Furthermore, understanding a patient’s drug metabolism is vital for preventing adverse reactions and optimizing therapeutic outcomes. Flexible SERS chips enable noninvasive, in situ monitoring of metabolites in biofluids like saliva, sweat, and urine, providing real-time insights into drug metabolism. For instance, the wearable plasmonic meta-surface sensor shown in Fig. 2C can track nicotine metabolism over time as shown in Fig. 4, illustrating its capability of assessing trace drug variations in the body and establish an individual’s metabolic profile [41]. Such monitoring facilitates dose adjustments to enhance efficacy and minimize side effects, essential for personalized healthcare. However, further studies are required to understand the relationship between drug concentrations in the sub-epidermis and those in the blood or interstitial fluid. This relationship is essential for accurately interpreting the data from noninvasive sensors and ensuring the reliability of the monitoring system.

### Infection prevention and control: Rapid pathogen detection

Rapid and effective pathogen detection is essential for managing infectious diseases. For instance, for detection of COVID-19, SERS-based LFIA is a great approach, significantly enhancing sensitivity and reducing detection time. This approach efficiently identifies SARS-CoV-2 variants through specific protein receptors. The strip features a 2D black phosphorus substrate decorated with Ag nanoparticles, which provides a larger size, higher specific surface area, and superior mechanical flexibility compared to conventional colloidal Au materials. This design enables faster flow on the LFIA strip, achieving results in just 5 to 20 min, with a sensitivity 1,000 times greater than commercial LFIA strips, and a detection limit of 0.5 pg/ml and 100 copies/ml for the N-protein and SARS-CoV-2. Additionally, the platform demonstrates high specificity in double-blind experiments with various coronaviruses, respiratory viruses, and medications by easily modifying the antibodies on the LFIA strips [77]. This adaptability suggests that the SERS-LFIA platform has significant potential as a next-generation antigen detection technology.

For effective infectious disease control, the recognition of pathogen types is also essential. It is a good choice to directly identify the pathogen types according to the difference of surface substances and chemical compositions. A multilayered SERS substrate made from electrospun polyvinylidene fluoride (PVDF) nanofibers has been developed. The dense distribution of Au nanoparticles on the surface of PVDF fibers enhances SERS signals and provides preconcentration of bacterial cells due to the higher specific surface area and porous morphology. This SERS platform can clearly identify *Escherichia coli* and *Staphylococcus aureus* by their spectral differences from the distinct protein compositions, providing great significance for quickly identifying the source of infection and implementing precise prevention and control [78].

In summary, flexible SERS chips hold great promise for personalized healthcare monitoring applications; especially, its specific recognition ability has a unique advantage for identifying unknown species, but further considerations are also needed for their real-life implementation. To ensure accurate results, the chip design must address the challenges posed by biomarker detection in biological fluids like blood, sweat, and urine, which can be influenced by environmental factors and individual variability. Integrating stimulus units, such as temperature control or drug release mechanisms, could help regulate or stimulate sweat production, improving the consistency of biomarker collection. Additionally, the accumulation of analytes can lead to signal saturation, reducing sensitivity and hindering continuous monitoring, which can be solved by separating the collection and detection units, allowing for fixed detection quantities and enabling continuous monitoring by replacing the detection unit. Another approach to maintaining long-term functionality involves the integration of self-cleaning mechanisms, such as hotspot erasure and reconstruction, to prevent unwanted adsorption of molecules.

## Miniaturized Spectrometers: Bringing Applications from Laboratory to Home

**Fig. 5.**
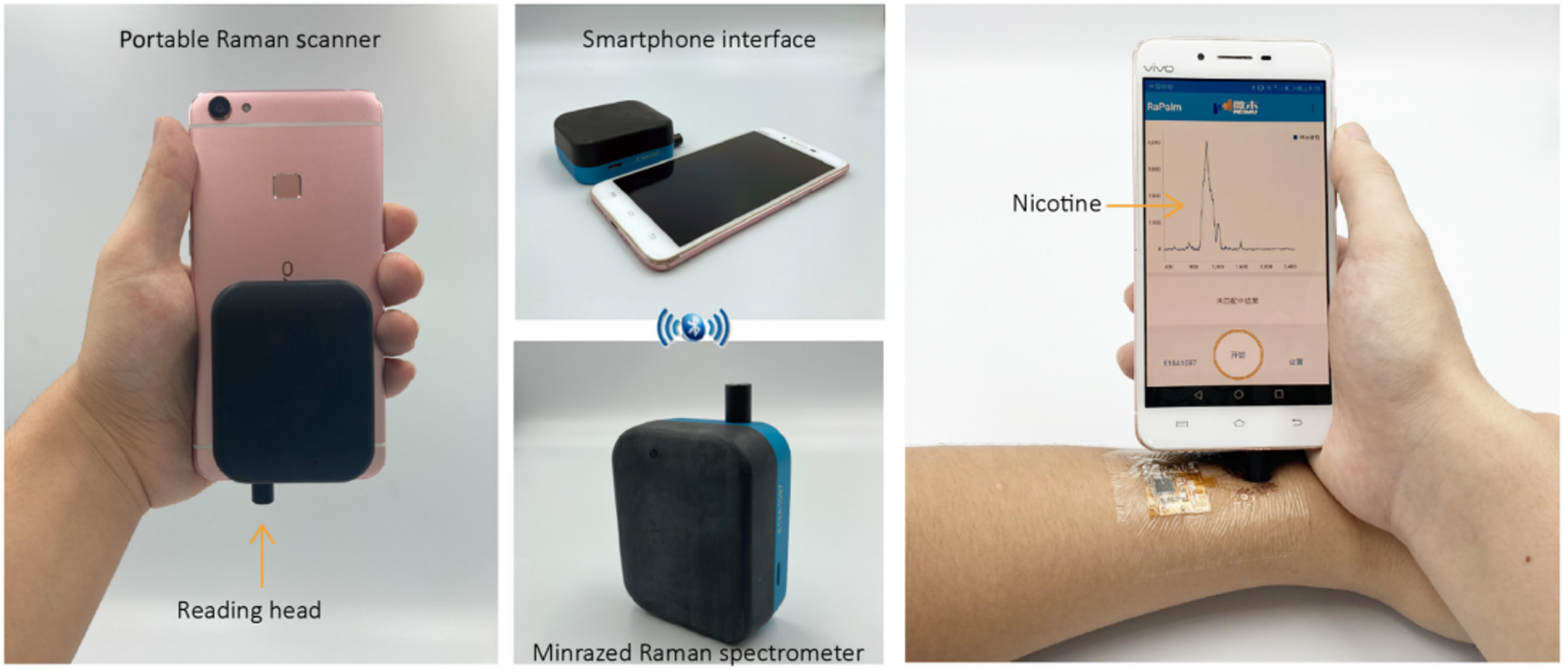
A proof-of-concept design for a miniaturized Raman spectrometer, built upon a commercial smartphone. The system integrates sensor control, Raman data acquisition, analysis, and sharing, all within a single app. Reproduced with permission from [41]. Copyright 2021, Science.

Flexible SERS chips exhibit the effective ability of sampling and detection for biomarker, driving their widespread adoption in personalized healthcare monitoring. The ultimate objective of personalized health monitoring is to facilitate reliable, convenient home testing, reducing reliance on laboratory-based diagnostics. Achieving this requires the miniaturization of detection devices to integrate seamlessly into everyday settings. Recent advancements in portable Raman spectrometers have addressed many of the critical requirements for SERS detection in biomedical applications, as depicted in Fig. 5 [37]. However, challenges such as high production costs and device size currently limit their broader application in routine home testing. Encouragingly, breakthroughs in nanotechnology, photonics, and microfabrication offer promising avenues for developing more compact, affordable, and wearable spectrometers. These innovations have the potential to revolutionize the accessibility of SERS-based diagnostics for home use. The following sections will delve into the key considerations and technological advancements driving the miniaturization of Raman spectrometers, paving the way for widespread deployment in personalized healthcare.

### Miniaturized laser source

Traditional Raman spectrometers typically rely on bulky gas lasers like argon-ion or krypton-ion, which are unsuitable for portable devices due to their size, weight, and power consumption. The shift to solid-state lasers, especially diode lasers, represents a significant advancement. Diode lasers are smaller, more efficient, and consume less power than gas lasers, making them easier to integrate into compact wearable systems while still providing the required wavelength stability and power for Raman scattering.

### Miniaturized detectors

Detectors are crucial in Raman spectrometers for capturing weak Raman signals. Traditional options like photomultiplier tubes are impractical for portable use due to their size, fragility, and power requirements. In contrast, solid-state detectors such as charge-coupled devices (CCDs) and complementary metal-oxide semiconductor (CMOS) sensors enable significant miniaturization. CCDs are effective for weak signal detection due to high quantum efficiency and low noise, while CMOS sensors offer faster readout speeds and easier integration with electronics, making them ideal for real-time analysis in portable devices. Although CMOS arrays typically have lower quantum efficiency than CCDs, their compact size and affordability support the miniaturization of Raman spectrometers and expand consumer markets for wearable biomedical and chemical sensing [79].

### Miniaturized optical components

Miniaturizing the optical components of a Raman spectrometer presents a critical challenge, influencing its resolution, spectral range, and signal-to-noise ratio (SNR). As the size decreases, aligning these components becomes more difficult, and stray light and fluorescence background increase, degrading performance [80]. According to the spectrometer design principles [81], effective miniaturization requires a focus on throughput, which is the product of the limiting area and the solid angle collected. A key challenge is to ensure that sufficient scattered Raman photons reach the detector for an adequate SNR. Currently, portable Raman spectrometers typically offer a resolution of about 9 cm^−1^ and a spectral range of 3,000 cm^−1^, with volumes from 200 cm^3^ to 1,700 cm^3^ and weights between 400 and 1,800 g [80]. To further reduce size, integration with smartphones is a promising approach, resulting in dimensions of 6.3 × 3.9 × 1.7 cm, a volume of about 42 cm^3^, and a weight of 63 g, while maintaining a spectral range of 400 to 2,300 cm^−1^ and a resolution of 16 to 19 cm^−1^.

However, these Raman spectrometers are all available commercial products, which are mostly based on well-established dispersive optics or Fourier transform (FT) techniques, and do not involve new dispersion technologies. To reduce the size of the Raman spectrometer to the millimeter scale, new methods need to be adopted:

1. Spectrometer based on dispersive optics. The incident optical spectra are split spatially and the individual channel intensity are measured. For this situation, the effective miniaturization methods include shortening or simplifying optical path, and utilizing micro-nano optics to reduce component size. Examples include using concave gratings, Grating–Fresnel lenses, and other diffractive elements [82,83]. It is important to note that according to the grating’s resolution formula λ/Δλ=KN (*N* is the total grooves on the grating), reducing size inevitably decreases spectral resolution and performance.

2. Spectrometer based on narrowband filters. A filter positioned in front of the detector achieves wavelength resolution by allowing the incident spectrum to pass through an adjustable filter or a filter array [84]. Narrowband filter-type spectrometers eliminate the need for bulky dispersion optics, allowing for a compact design by placing the filter very close to the detector [85]. However, when measuring broadband light, the transmission properties of narrowband filters must be adjusted either temporally or spatially [86]. While optical filter-based spectrometers offer compactness suitable for space-constrained applications, this comes at the expense of longer measurement times and reduced spectral resolution.

3. Spectrometers based on FT. FT spectrometers, commonly used for infrared absorption or emission spectra, employ an interferometer to modulate incident light on a single detector over time. The resulting “interferogram” is converted into a wavelength-dependent spectrum via FT. Spectral resolution and bandwidth are determined by the maximum optical path difference (OPD) and the sampling interval of the OPD. FT spectrometers, lacking slits or apertures, benefit from high optical throughput (Jacquinot advantage) and multiple channels (Fellgett advantage), offering a higher SNR, compact size, and lower cost compared to dispersive spectrometers [87]. However, micro-Fourier spectrometers often depend on electromagnetic or electro-thermal micro-electromechanical systems for mirror movements, which can limit stroke and spectral resolution [88]. Recently, a spatial heterodyne Fourier Raman spectrometer was introduced without movable components, measuring just 3.5 × 3.5 × 2.5 cm, weighing about 80 g, with a spectral range of approximately 3,500 cm^−1^ and a resolution of 4 to 5 cm^−1^, indicating promising potential for miniaturization [89].

4. Spectrometer based on reconstructive spectrum. Dispersive spectrometers typically use optics like gratings or prisms to achieve wavelength resolution, making it challenging to enhance this resolution within a limited optical path. However, wavelength dispersion is not essential for all spectrometers. Instead, spectra can be reconstructed by sampling the coefficients of incident light at each wavelength point. This principle can be mathematically explained, illustrating that by capturing sufficient data points across the spectrum, it is possible to derive a complete spectral profile without relying on traditional dispersive methods. When a signal with a spectrum *s*(*λ*) propagates through a normalized signature patterns *p*(*λ*, *x*), so the signal at the detector array is measured as *I*(*x*), the mathematical relation can be described as $I\left(x\right)=\int_{\lambda 1}^{\lambda 2}p\left(\lambda ,\;x\right)\cdot s\left(\lambda \right)\mathrm{d\lambda}$. As long as the *I*(*x*) and *p*(*λ*, *x*) are known, *s*(*λ*) can be reconstructed. Reconstructive spectrum gives a new way to further reduce spectrometers size and cost, which is likely to be a feasible solution for miniaturized Raman spectrometer compared with the first 3 routes, with no breakthroughs in fundamental technology, concepts, and industrialization [90,91].

### Challenges and opportunities of miniaturized spectrometer

It is worth emphasizing that Ilchenko and his team developed a design that significantly reduces the hardware requirements for spectrometers [92]. By comparing signals from a specimen and a standard sample in real time, they calibrate signal drift from environmental instability and excitation light fluctuations. This results in a smaller, lower-cost Raman spectrometer with dimensions of 7 × 2 × 0.8 cm, a spectral range of 400 to 4,000 cm^−1^, a resolution of 7 cm^−1^, and a power consumption of about 2 W, making it suitable for field applications, where size, weight, and power consumption are critical constraints.

Moreover, integrating AI with Raman spectrometers significantly enhances their functionality by improving signal acquisition, reducing noise, and increasing resolution. Additionally, researchers are increasingly combining micro-spectrometer devices with everyday smart devices, such as mobile phones and smart watches. This integration leverages the computational power, connectivity, and user-friendly interfaces of these devices, enabling seamless operation and data visualization. Such combinations reduce the overall size and complexity of spectrometers while enhancing their performance and accessibility. For instance, a Raman spectrometer paired with a smartphone could provide on-the-go health insights, transmitting data to healthcare providers or cloud platforms for advanced analysis and storage. This trend of miniaturization, coupled with AI-driven advancements, holds the promise of transforming healthcare by bringing laboratory-level diagnostics into homes. It is expected to support the development of portable diagnostic kits, which enables continuous personalized health monitoring, offering timely and actionable insights into health conditions.

## AI: Empowering SERS Data Processing and Analysis

While flexible SERS chips have considerable potential for personalized healthcare monitoring, challenges remain in rapidly and accurately extracting meaningful data from complex biological samples. SERS generates high-dimensional data that reflect intricate molecular details, yet interpreting this data to identify specific biomarkers or distinguish health states often exceeds traditional analytical methods. Biological samples can also present complex signals, low SNRs, and fluorescence interference, complicating both qualitative and quantitative analyses.

Integrating AI effectively addresses these challenges by enhancing data acquisition, processing, and analysis [93,94]. Advances in machine learning (ML) and natural language processing (NLP) enable AI to identify complex patterns in extensive datasets [95–97]. In the context of SERS, multivariate statistical methods and both supervised or unsupervised ML algorithms facilitate the detection of subtle spectral features, improving interpretation and classification [98,99]. Deep learning architectures, such as CNNs and RNNs, further enhance AI’s capacity to manage large datasets, supporting advanced feature extraction and pattern recognition while minimizing overfitting [100,101]. By leveraging the high sensitivity and specificity of SERS, AI significantly improves biomarker detection and classification, addressing issues like low SNRs and fluorescence interference, thus advancing personalized health monitoring [102].

### Data processing and enhancement

Effective data processing is crucial for enhancing the performance of SERS in personalized healthcare monitoring. By addressing issues such as cosmic ray interference, low SNR, and baseline drift, data processing ensures cleaner and more reliable spectral data for analysis. Traditional methods, such as polynomial fitting and manual adjustments, often fall short when dealing with the complexities of biological or environmental samples. In contrast, AI-driven techniques offer automated, efficient, and precise solutions, significantly improving the usability and accuracy of SERS in healthcare applications.

For instance, CNNs have been applied to low-SNR Raman spectra, such as those from prostate cancer cell lines. Through customized loss functions, CNNs effectively eliminate random noise and correct baseline drift, preserving key spectral features [103]. Furthermore, CNNs can autonomously identify and differentiate cosmic ray artifacts from Raman peaks. By training on datasets containing spectra with spikes and corresponding reference spectra without spikes, CNNs achieve accurate detection and removal of cosmic rays, regardless of their intensity or overlap with Raman peaks [104]. This capability minimizes human intervention and enhances data reliability. Denoising autoencoders (DAs) provide another robust approach to improving SNR. By learning and removing noise patterns during spectral reconstruction, DAs have been shown to significantly enhance SNR, increasing it from 4.1 to 7.2 for 0.1 s/cell spectra and from 8.0 to 17.0 for 1 s/cell spectra. This advancement has facilitated rapid and accurate single-cell diagnosis of bacterial pathogens, demonstrating the potential of DAs in high-speed, high-precision analyses [105].

**Fig. 6.**
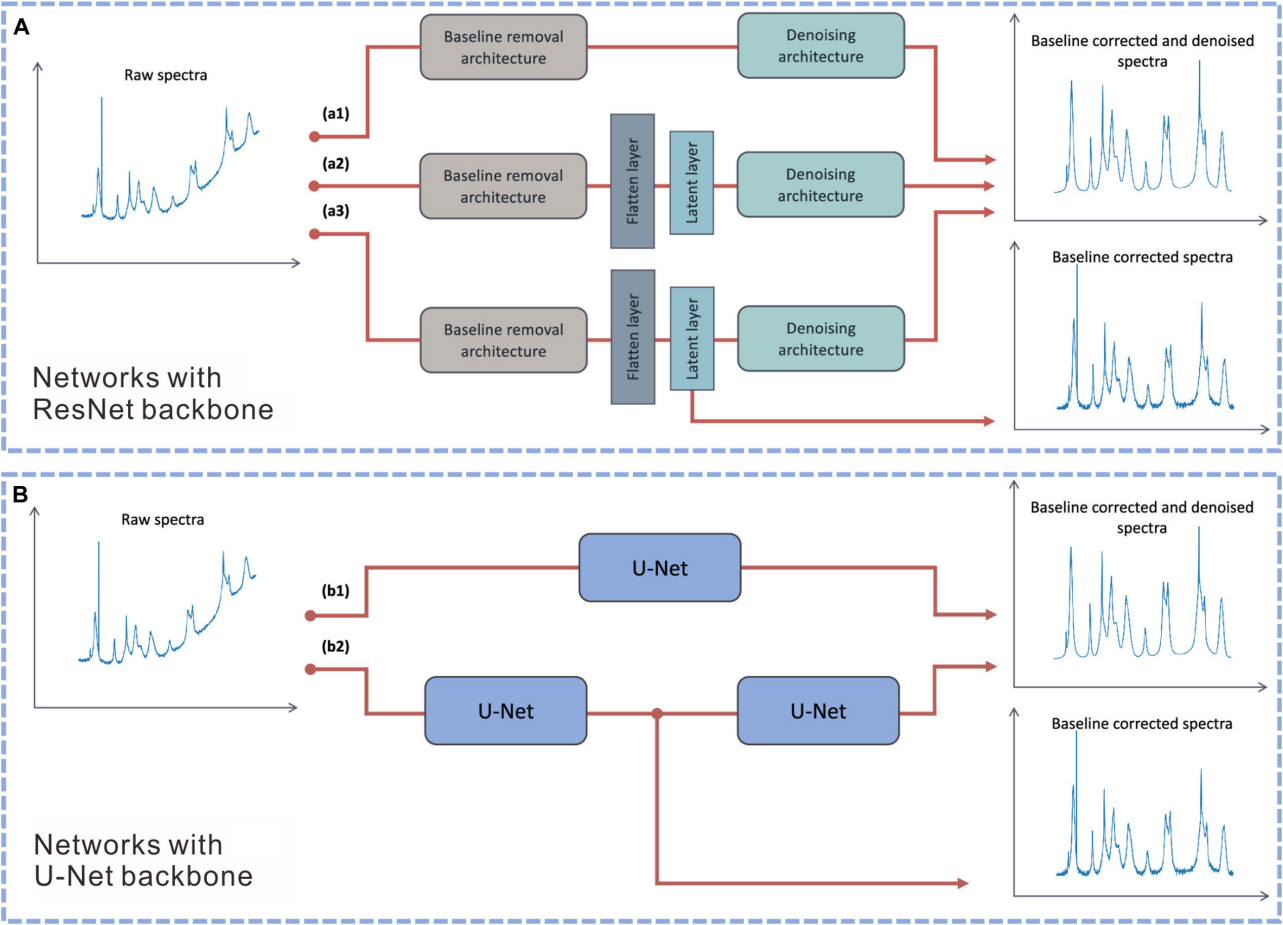
Baseline correction and denoising of SERS spectra via cascaded deep convolutional neural network (CNN) architectures. (A) Based on ResNet, demonstrating various spectral preprocessing steps: (a1) baseline correction followed by noise removal, (a2) baseline correction terminating at the latent layer before denoising, and (a3) latent layer output trained using noisy signals. (B) Based on U-Net, illustrating 2 architectures: (b1) single U-Net for single-step spectral preprocessing, and (b2) cascaded U-Net for multistep spectral preprocessing. Reproduced with permission from [106]. Copyright 2022, American Chemical Society.

Baseline correction is another critical aspect of data processing. Traditional techniques, such as polynomial fitting and adaptive iteratively reweighted penalized least squares, estimate and subtract background contributions to refine the spectra. However, advanced deep learning methods, particularly U-Net architectures, have introduced more sophisticated and automated approaches. For example, as illustrated in Fig. 6, cascaded CNN systems integrating U-Net and ResNet architectures autonomously preprocess raw data, delivering both denoised and baseline-corrected spectra. These systems outperform conventional methods like Savitzky–Golay filters and wavelet transforms, achieving faster denoising, reducing mean squared error (MSE) by 50%, and identifying broad background signals and narrow spectral peaks in a single step [106].

### Feature extraction and identification

AI-assisted SERS revolutionizes disease diagnosis by providing precise biomarker analysis, offering insights into disease status and progression. Biomarkers such as PSA serve as essential tools for cancer detection and monitoring. Leveraging advanced AI models, such as PLS–support vector machines (SVMs), researchers analyzed SERS spectra of PSA from both healthy individuals and prostate cancer patients. This method demonstrated high diagnostic accuracy, achieving a 95% success rate in distinguishing between healthy volunteers and prostate cancer patients. The AI-driven model not only simplifies the detection process but also improves reliability by reducing variability in spectral data [107].

Beyond specific biomarkers, AI-assisted SERS extends its utility to other diseases by identifying subtle spectral variations linked to metabolic dysregulation. For instance, in conditions like Sjögren’s syndrome and diabetic nephropathy, while characteristic peak positions remain stable, variations in intensity reflect underlying metabolic shifts. Principal components analysis (PCA) is instrumental in extracting multidimensional spectral features, forming a foundation for classification using SVMs. This approach has achieved identification accuracies of 90.1% for Sjögren’s syndrome and 89.3% for diabetic nephropathy, demonstrating its effectiveness in differentiating complex disease profiles [108].

**Fig. 7.**
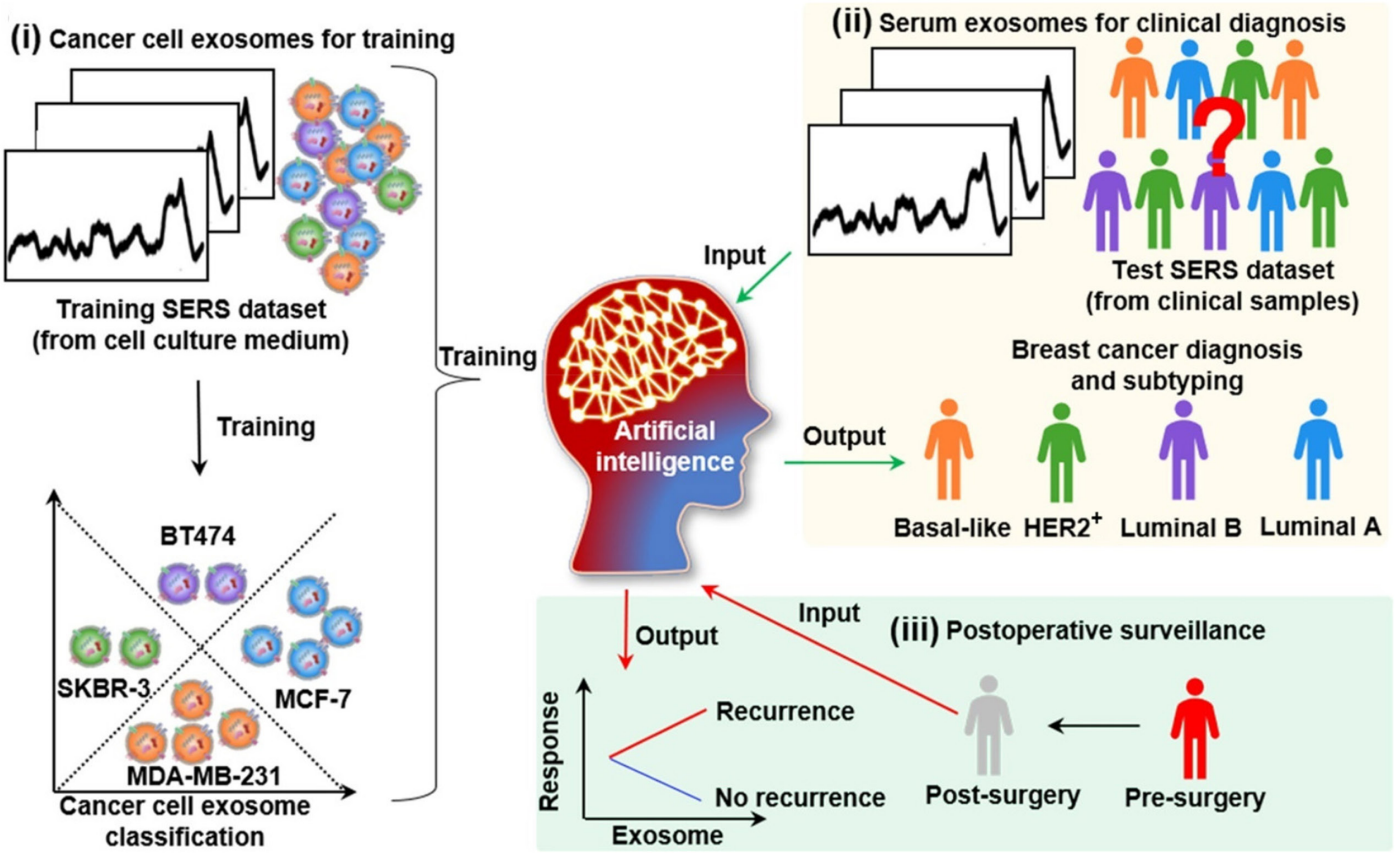
Feature extraction and disease identification via deep learning-assisted SERS analysis. Application of artificial neural network (ANN) algorithms to extract information from SERS data from plasma exosomes for accurate diagnosis of breast cancer and assessment of surgical outcomes. Reproduced with permission from [109]. Copyright 2022, American Chemical Society.

Neural networks, such as artificial neural networks (ANNs), effectively capture complex relationships within spectral data, advancing diagnostic precision. For example, ANNs have been applied to label-free SERS analysis of plasma exosomes for breast cancer diagnosis and surgical outcome evaluation, as shown in Fig. 7. Breast cancer exosomes exhibit distinct SERS spectral patterns that correlate with cancer subtypes and progression stages. Training ANNs on SERS datasets derived from exosomes of different breast cancer cell lines enables accurate detection and therapeutic assessment. This technique has proven instrumental in diagnosing breast cancer and monitoring treatment efficacy [109].

Similarly, ANN-assisted SERS has shown promise in brain cancer prognosis. By analyzing the spectral signatures of molecules such as glycogen, phosphatidylinositol, nucleic acids, and lipids, researchers achieved a diagnostic accuracy of 96% in differentiating between primary and metastatic brain tumors. Furthermore, the method accurately identified tumor locations, demonstrating its potential to guide surgical interventions and treatment planning [110].

### Pattern recognition and classification

Diseases often generate unique spectral patterns and specific peaks in SERS spectra, providing a basis for differentiation. Spectral overlap among analytes presents a considerable challenge in SERS applications, as it complicates the accurate identification of target molecules. Advanced multivariate analysis methods applied to baseline-corrected SERS data enable the effective identification and classification of these disease-specific patterns.

For example, urine samples analyzed using a combination of unsupervised PCA and supervised orthogonal partial least squares discriminant analysis (OPLS-DA) demonstrated exceptional diagnostic performance. The analysis was conducted on a 3D-stacked silver nanowire sensor integrated with a glass fiber filter, enhancing sensitivity and reproducibility. This approach successfully distinguished between pancreatic and prostate cancer groups, achieving 100% sensitivity and specificity. Furthermore, it effectively separated normal controls from cancer patients, maintaining the same high sensitivity and specificity [111].

**Fig. 8.**
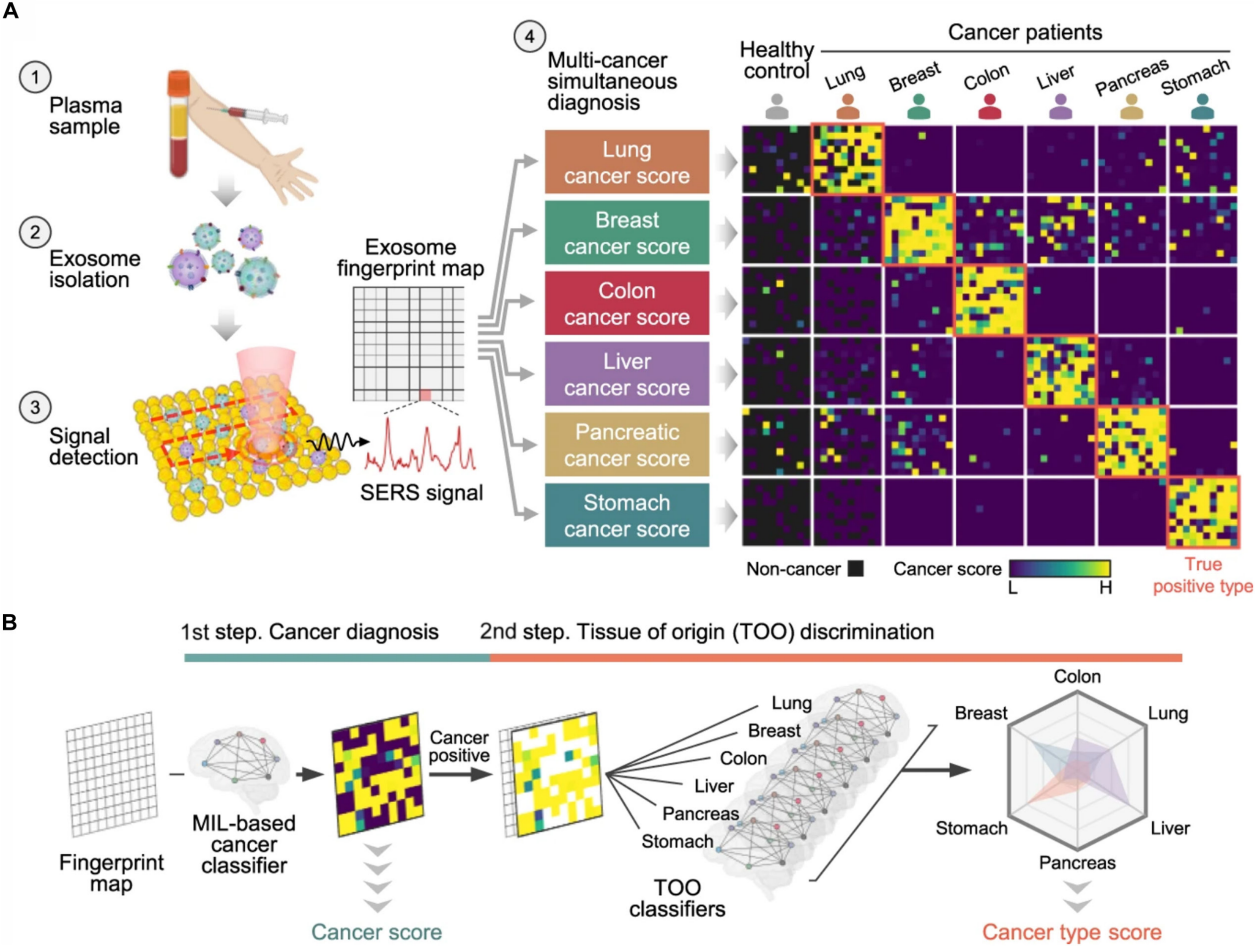
Cancer recognition and classification via AI algorithms. (A) Exosome suspensions are applied to an Au nanoparticle array chip, and Raman signals are collected from 100 spots in a 10 × 10 grid. The AI system then predicts cancer presence and identifies the tissue of origin. A heat map illustrates representative outcomes for various cancer statuses. (B) The AI-based diagnostic workflow begins with the calculation of diagnostic scores using multiple instance learning (MIL)-based classifiers. In the second step, the predicted signals are further analyzed, and a final score is computed by integrating results from 6 different prediction models. Reproduced with permission from [112]. Copyright 2023, Nature.

Additionally, integrating deep learning with label-free SERS analysis of plasma exosomes demonstrates wide diagnostic applicability. This method effectively distinguishes between 6 early-stage cancer types, lung, breast, colon, liver, pancreas, and stomach, with a sensitivity of 90.2% and a specificity of 94.4%. Notably, it accurately predicts the tumor organ in 72% of positive cases. By utilizing nonspecific Raman signatures, this approach shows great potential for broadening its diagnostic capabilities to encompass other diseases, as shown in Fig. 8 [112].

Other key challenge in diagnostics is differentiating between diseases that share biomarkers, such as lung cancer and gastric cancer, both linked to aldehydes exhaled breath. To tackle this, a deep learning-based ANN was developed and trained on a comprehensive dataset of SERS spectra from healthy individuals and patients with lung cancer and gastric cancer. The model achieved over 89% accuracy in distinguishing between the 2 cancers. Moreover, by mining SERS peak data, the ANN identified subtle compositional differences in the breath of healthy individuals versus cancer patients, thereby enhancing diagnostic precision [113].

Moreover, to tackle challenges such as spectral overlap, labeling methods can be utilized, enhancing SERS substrates with multiple gene probes for cancer cell mutations, each tagged with unique Raman reporters. This facilitates simultaneous detection of various mutations through spectral decoding. By clustering SERS spectra from mutation assays, supervised algorithms like classical least squares (CLS) combined with linear discriminant analysis (LDA) can effectively classify cancer types, successfully differentiating colorectal cancer from melanoma within 40 min with over 90% accuracy [114].

To conclude, the integration of AI with SERS technology offers transformative potential for personalized healthcare, enhancing precision in data analysis and biomarker detection. AI-driven approaches enable real-time health monitoring, fostering timely interventions and proactive, tailored care. However, challenges remain, including the need for robust train data that generalize across diverse populations and account for environmental and sample variability. Future efforts should prioritize advancing AI algorithms for greater accuracy, scalability, and reliability, alongside optimizing SERS chip designs and quantification ability to enhance sensitivity and selectivity for specific biomarkers. The synergy between AI and SERS holds significant promise for the future of personalized health diagnostics.

## Conclusion and Outlook

This review systematically summarizes the significant promise for the integration of SERS into personalized healthcare. The advancements in flexible SERS chip design and fabrication have paved the way for more practical applications of personalized healthcare monitoring. These flexible SERS chips, with their improved target processing and detection, enable the ex vivo or in situ detection of most biological markers, which is crucial for chronic disease management, disease diagnosis, drug safety monitoring, as well as prevention and control of infectious diseases. The development of miniaturized spectrometers has further enhanced the applicability of SERS in POCT, providing immediate and reliable diagnostic information. Moreover, the integration of AI in SERS data processing and analysis is revolutionizing the field. Consequently, through the coordination of these aspects, SERS-based personalized health monitoring is expected to become a reality. Nevertheless, there are still several key points that need further research when looking forward:

1. Sensitivity and specificity: Ongoing efforts to improve the sensitivity and specificity of SERS chips will be crucial. This includes exploring novel nanomaterials and optimizing fabrication techniques to achieve more consistent and reliable results. SERS-active materials, including 2D materials and other new SERS materials, should be paid attention to, because of the unique specificity and better stability over conventional noble metals.

2. Qualitative and quantification: SERS technology offers exceptional molecular recognition capabilities, but its application in personalized health monitoring faces several challenges. One major issue is the complexity of biological samples needed to establish large databases to match specific targets. Moreover, quantitative detection remains a significant barrier to broader SERS adoption. Despite advancements, such as optimized substrate preparation, the use of internal standards, and the increasingly discussed chemometric techniques, achieving reliable and precise quantification in SERS continues to be challenging [115,116]. Further innovation in developing accurate and efficient quantitative methods is crucial to unlocking the full potential of SERS for real-world applications in healthcare monitoring.

3. Real-time monitoring: The development of SERS platforms for real-time monitoring of biomarkers holds immense promise for the future of personalized healthcare. These advanced systems will enable timely medical interventions, allowing healthcare providers to respond rapidly to changes in a patient’s condition. Continuous analysis of biological samples can provide instant feedback on health status, facilitating dynamic treatment adjustments tailored to individual needs. As technology advances, integrating these platforms with AI and ML will enhance predictive capabilities, enabling more proactive and personalized approaches to disease management. Ultimately, this could lead to improved patient outcomes and a shift toward preventive care models in healthcare.

4. Cost: Efforts to reduce the cost of SERS substrates and portable spectrometers are crucial for enhancing the accessibility of these technologies, especially in low-resource settings. By making SERS more affordable, healthcare providers in underserved areas can implement advanced diagnostic tools that facilitate personalized medicine. This accessibility will empower clinicians to monitor disease biomarkers in real time, leading to timely interventions and improved patient outcomes. Furthermore, widespread use of cost-effective SERS technology could support public health initiatives by enabling early detection of diseases and fostering proactive health management strategies.

5. Ethical and privacy considerations: As AI integrates more deeply with SERS data analysis, addressing ethical and privacy concerns surrounding patient data will be essential. Ensuring robust data security measures and obtaining informed patient consent will be critical to maintaining trust and compliance with regulations. This focus on ethics will help facilitate the responsible adoption of AI-enhanced SERS technologies in healthcare.

## References

[1] Libanori A, Chen G, Zhao X, Zhou Y, Chen J. Smart textiles for personalized healthcare. Nat Electron. 2022;5:142–156. 10.1038/s41928-022-00723-z PMC13309183 42367254

[2] Johnson KB, Wei W-Q, Weeraratne D, Frisse ME, Misulis K, Rhee K, Zhao J, Snowdon JL. Precision medicine, AI, and the future of personalized health care. Clin Transl Sci. 2021;14(1):86–93. 32961010 10.1111/cts.12884 PMC7877825

[3] Son D, Lee J, Qiao S, Ghaffari R, Kim J, Lee JE, Song C, Kim SJ, Lee DJ, Jun SW, et al. Multifunctional wearable devices for diagnosis and therapy of movement disorders. Nat Nanotechnol. 2014;9(5):397–404. 24681776 10.1038/nnano.2014.38

[4] Yokota T, Fukuda K, Someya T. Recent progress of flexible image sensors for biomedical applications. Adv Mater. 2021;33(19):2004416. 10.1002/adma.202004416 33527511

[5] Park S, Won DD, Lee BJ, Escobedo D, Estevas A, Aalipour A, Ge TJ, Kim JH, Suh S, Choi EH, et al. A mountable toilet system for personalized health monitoring via the analysis of excreta. Nat Biomed Eng. 2020;4(6):624–635. 32251391 10.1038/s41551-020-0534-9 PMC7377213

[6] Min J, Tu J, Xu C, Lukas H, Shin S, Yang Y, Solomon SA, Mukasa D, Gao W. Skin-interfaced wearable sweat sensors for precision medicine. Chem Rev. 2023;123(8):5049–5138. 36971504 10.1021/acs.chemrev.2c00823 PMC10406569

[7] Cutshaw G, Uthaman S, Hassan N, Kothadiya S, Wen X, Bardhan R. The emerging role of Raman spectroscopy as an omics approach for metabolic profiling and biomarker detection toward precision medicine. Chem Rev. 2023;123(13):8297–8346. 37318957 10.1021/acs.chemrev.2c00897 PMC10626597

[8] Ji W, Zhu J, Wu W, Wang N, Wang J, Wu J, Wu Q, Wang X, Yu C, Wei G, et al. Wearable sweat biosensors refresh personalized health/medical diagnostics. Research. 2021;2021: Article 9757126. 34778790 10.34133/2021/9757126 PMC8557357

[9] Sempionatto JR, Lin M, Yin L, de la Paz E, Pei K, Sonsa-ard T, de Loyola Silva AN, Khorshed AA, Zhang F, Zhang F, et al. An epidermal patch for the simultaneous monitoring of haemodynamic and metabolic biomarkers. Nat Biomed Eng. 2021;5(7):737–748. 33589782 10.1038/s41551-021-00685-1

[10] Kim D-H, Cha J-H, Lim JY, Bae J, Lee W, Yoon KR, Kim C, Jang JS, Hwang W, Kim ID. Colorimetric dye-loaded nanofiber yarn: Eye-readable and weavable gas sensing platform. ACS Nano. 2020;14(12):16907–16918. 33275412 10.1021/acsnano.0c05916

[11] Li C, Chen G, Zhang Y, Wu F, Wang Q. Advanced fluorescence imaging technology in the near-infrared-II window for biomedical applications. J Am Chem Soc. 2020;142(35):14789–14804. 32786771 10.1021/jacs.0c07022

[12] Ding S-Y, Yi J, Li J-F, Ren B, Wu D-Y, Panneerselvam R, Tian ZQ. Nanostructure-based plasmon-enhanced Raman spectroscopy for surface analysis of materials. Nat Rev Mater. 2016;1:16021.

[13] Langer J, Jimenez De Aberasturi D, Aizpurua J, Alvarez-Puebla RA, Auguié B, Baumberg JJ, Bazan GC, Bell SEJ, Boisen A, Brolo AG, et al. Present and future of surface-enhanced Raman scattering. ACS Nano. 2020;14(1):28–117. 31478375 10.1021/acsnano.9b04224 PMC6990571

[14] Tang X, Hao Q, Hou X, Lan L, Li M, Yao L, Zhao X, Ni Z, Fan X, Qiu T. Exploring and engineering 2D transition metal dichalcogenides toward ultimate SERS performance. Adv Mater. 2024;36(19):2312348. 10.1002/adma.202312348 38302855

[15] Hao Q, Peng Z, Wang J, Fan X, Li G, Zhao X, Ma L, Qiu T, Schmidt OG. Verification and analysis of single-molecule SERS events via polarization-selective Raman measurement. Anal Chem. 2022;94(2):1046–1051. 34989240 10.1021/acs.analchem.1c04015

[16] Zhao X, Hao Q, Ni Z-H, Qiu T. Single-molecule surface-enhanced Raman spectroscopy (SM-SERS): Characteristics and analysis. Acta Phys Sin. 2021;70(13): Article 137401.

[17] Lee W, Kang B-H, Yang H, Park M, Kwak JH, Chung T, Jeong Y, Kim BK, Jeong KH. Spread spectrum SERS allows label-free detection of attomolar neurotransmitters. Nat Commun. 2021;12(1):159. 33420035 10.1038/s41467-020-20413-8 PMC7794485

[18] Shan B, Pu Y, Chen Y, Liao M, Li M. Novel SERS labels: Rational design, functional integration and biomedical applications. Coord Chem Rev. 2018;371:11–37.

[19] Plou J, Valera PS, García I, de Albuquerque CDL, Carracedo A, Liz-Marzán LM. Prospects of surface-enhanced Raman spectroscopy for biomarker monitoring toward precision medicine. ACS Photonics. 2022;9(2):333–350. 35211644 10.1021/acsphotonics.1c01934 PMC8855429

[20] Moore TL, Rodriguez-Lorenzo L, Hirsch V, Balog S, Urban D, Jud C, Rothen-Rutishauser B, Lattuada M, Petri-Fink A. Nanoparticle colloidal stability in cell culture media and impact on cellular interactions. Chem Soc Rev. 2015;44(17):6287–6305. 26056687 10.1039/c4cs00487f

[21] Lee S, Dang H, Moon J-I, Kim K, Joung Y, Park S, Yu Q, Chen J, Lu M, Chen L, et al. SERS-based microdevices for use as in vitro diagnostic biosensors. Chem Soc Rev. 2024;53(11):5394–5427. 38597213 10.1039/d3cs01055d

[22] Cialla-May D, Bonifacio A, Bocklitz T, Markin A, Markina N, Fornasaro S, Dwivedi A, Dib T, Farnesi E, Liu C, et al. Biomedical SERS—The current state and future trends. Chem Soc Rev. 2024;53(18):8957–8979. 39109571 10.1039/d4cs00090k

[23] Xu K, Zhou R, Takei K, Hong M. Toward flexible surface-enhanced Raman scattering (SERS) sensors for point-of-care diagnostics. Adv Sci. 2019;6(16):1900925. 10.1002/advs.201900925 PMC6702763 31453071

[24] Liu X, Guo J, Li Y, Wang B, Yang S, Chen W, Wu X, Guo J, Ma X. SERS substrate fabrication for biochemical sensing: Towards point-of-care diagnostics. J Mater Chem B. 2021;9(10):8378–8388. 34505606 10.1039/d1tb01299a

[25] Liu G, Mu Z, Guo J, Shan K, Shang X, Yu J, Liang X. Surface-enhanced Raman scattering as a potential strategy for wearable flexible sensing and point-of-care testing non-invasive medical diagnosis. Front Chem. 2022;10:1060322. 36405318 10.3389/fchem.2022.1060322 PMC9669362

[26] Lee CH, Tian L, Singamaneni S. Paper-based SERS swab for rapid trace detection on real-world surfaces. ACS Appl Mater Interfaces. 2010;2(12):3429–3435. 21128660 10.1021/am1009875

[27] Lan L, Fan X, Yu S, Gao J, Zhao C, Hao Q, Qiu T. Flexible two-dimensional vanadium carbide MXene-based membranes with ultra-rapid molecular enrichment for surface-enhanced Raman scattering. ACS Appl Mater Interfaces. 2022;14(35):40427–40436. 35998890 10.1021/acsami.2c10800

[28] Wang L, Wang X, Cheng L, Ding S, Wang G, Choo J, Chen L. SERS-based test strips: Principles, designs and applications. Biosens Bioelectron. 2021;189: Article 113360. 34051383 10.1016/j.bios.2021.113360

[29] Chen S, Meng L, Wang L, Huang X, Ali S, Chen X, Yu M, Yi M, Li L, Chen X, et al. SERS-based lateral flow immunoassay for sensitive and simultaneous detection of anti-SARS-CoV-2 IgM and IgG antibodies by using gap-enhanced Raman nanotags. Sensors Actuators B Chem. 2021;348: Article 130706. 10.1016/j.snb.2021.130706 PMC8413105 34493903

[30] Liang P, Guo Q, Zhao T, Wen C-Y, Tian Z, Shang Y, Xing J, Jiang Y, Zeng J. Ag nanoparticles with ultrathin Au shell-based lateral flow immunoassay for colorimetric and SERS dual-mode detection of SARS-CoV-2 IgG. Anal Chem. 2022;94(23):8466–8473. 35657150 10.1021/acs.analchem.2c01286

[31] Liu H, Dai E, Xiao R, Zhou Z, Zhang M, Bai Z, Shao Y, Qi K, Tu J, Wang C, et al. Development of a SERS-based lateral flow immunoassay for rapid and ultra-sensitive detection of anti-SARS-CoV-2 IgM/IgG in clinical samples. Sensors Actuators B Chem. 2021;329: Article 129196. 10.1016/j.snb.2020.129196 PMC7673228 33230369

[32] Xiao R, Lu L, Rong Z, Wang C, Peng Y, Wang F, Wang J, Sun M, Dong J, Wang D, et al. Portable and multiplexed lateral flow immunoassay reader based on SERS for highly sensitive point-of-care testing. Biosens Bioelectron. 2020;168: Article 112524. 32866724 10.1016/j.bios.2020.112524

[33] Deng R, Xia Z, Yan F, Feng X, Zhang G, Li X. Inkjet printing patterned plasmonic SERS platform with surface-optimized paper for label-free detection of illegal drugs in urine. Anal Chem. 2024;96(42):16834–16841. 39373888 10.1021/acs.analchem.4c03549

[34] Restaino SM, White IM. A critical review of flexible and porous SERS sensors for analytical chemistry at the point-of-sample. Anal Chim Acta. 2019;1060:17–29. 30902328 10.1016/j.aca.2018.11.057

[35] Ansah IB, Kim S, Yang J-Y, Mun C, Jung HS, Lee S, Kim DH, Kim SH, Park SG. In situ electrodeposition of gold nanostructures in 3D ultra-thin hydrogel skins for direct molecular detection in complex mixtures with high sensitivity. Laser Photonics Rev. 2021;15(12):2100316.

[36] Gao T, Yachi T, Shi X, Sato R, Sato C, Yonamine Y, Kanie K, Misawa H, Ijiro K, Mitomo H. Ultrasensitive surface-enhanced Raman scattering platform for protein detection via active delivery to nanogaps as a hotspot. ACS Nano. 2024;18(32):21593–21606. 39093951 10.1021/acsnano.4c09578 PMC11328179

[37] Ding Q, Wang J, Chen X, Liu H, Li Q, Wang Y, Yang S. Quantitative and sensitive SERS platform with analyte enrichment and filtration function. Nano Lett. 2020;20(10):7304–7312. 32866018 10.1021/acs.nanolett.0c02683

[38] Shi Y, Fang J. Yolk–Shell hierarchical pore au@MOF nanostructures: Efficient gas capture and enrichment for advanced breath analysis. Nano Lett. 2024;24(33):10139–10147. 39109658 10.1021/acs.nanolett.4c02267

[39] Li Z, Huang X, Lu G. Recent developments of flexible and transparent SERS substrates. J Mater Chem C. 2020;8:3956–3969.

[40] Yao L, Hao Q, Li M, Fan X, Li G, Tang X, Wei Y, Wang J, Qiu T. Flexible plasmonic nanocavities: A universal platform for the identification of molecular orientations. Nanoscale. 2023;15(14):6588–6595. 36961297 10.1039/d3nr01059g

[41] Wang Y, Zhao C, Wang J, Luo X, Xie L, Zhan S, Kim J, Wang X, Liu X, Ying Y. Wearable plasmonic-metasurface sensor for noninvasive and universal molecular fingerprint detection on biointerfaces. Sci Adv. 2021;7(4):eabe4553. 33523953 10.1126/sciadv.abe4553 PMC10964967

[42] Lee W-C, Koh EH, Kim D-H, Park S-G, Jung HS. Plasmonic contact lens materials for glucose sensing in human tears. Sensors Actuators B Chem. 2021;344: Article 130297.

[43] Li G, Zhao X, Tang X, Yao L, Li W, Wang J, Liu X, Han B, Fan X, Qiu T, et al. Wearable hydrogel SERS chip utilizing plasmonic trimers for uric acid analysis in sweat. Nano Lett. 2024;24(42):13447–13454. 39392787 10.1021/acs.nanolett.4c04267

[44] Liu R, Nie Q, Wang Y, Wu Y, Tu Y, Xie C, Xiao X, You R, Lu Y. Diaper-based wearable SERS sensing system with a silver nano dual-structure composite hydrogel for the detection of biomarkers and pH in urine. Chem Eng J. 2024;498: Article 155207.

[45] Xing C, Luo M, Sheng Q, Zhu Z, Yu D, Huang J, He D, Zhang M, Fan W, Chen D. Silk fabric functionalized by nanosilver enabling the wearable sensing for biomechanics and biomolecules. ACS Appl Mater Interfaces. 2024;16(38):51669–51678. 39268841 10.1021/acsami.4c10253

[46] Lee H, Liao J-D, Tsai H-P, Chen C-H, Sitjar J, Fu W-E, Lin FH. Label-free SERS method with size-matched selectivity for analytes of varying sizes. Surf Interfaces. 2024;44: Article 103821.

[47] Chung M, Skinner WH, Robert C, Campbell CJ, Rossi RM, Koutsos V, Radacsi N. Fabrication of a wearable flexible sweat pH sensor based on SERS-active Au/TPU electrospun nanofibers. ACS Appl Mater Interfaces. 2021;13(43):51504–51518. 34672514 10.1021/acsami.1c15238

[48] Liu L, Martinez Pancorbo P, Xiao T, Noguchi S, Marumi M, Segawa H, Karhadkar S, Gala de Pablo J, Hiramatsu K, Kitahama Y, et al. Highly scalable, wearable surface-enhanced Raman spectroscopy. Adv Opt Mater. 2022;10(17):2200054.

[49] Guo J, Zeng F, Guo J, Ma X. Preparation and application of microfluidic SERS substrate: Challenges and future perspectives. J Mater Sci Technol. 2020;37:96–103.

[50] Wang J, Kao Y-C, Zhou Q, Wuethrich A, Stark MS, Schaider H, Soyer HP, Lin LL, Trau M. An integrated microfluidic-SERS platform enables sensitive phenotyping of serum extracellular vesicles in early stage melanomas. Adv Funct Mater. 2022;32(3):2010296.

[51] He X, Fan C, Luo Y, Xu T, Zhang X. Flexible microfluidic nanoplasmonic sensors for refreshable and portable recognition of sweat biochemical fingerprint. npj Flex Electron. 2022;6:60.

[52] Ye H, Chen X, Huang X, Li C, Yin X, Zhao W, Wang T. Patterned gold nanoparticle superlattice film for wearable sweat sensors. Nano Lett. 2024;24(35):11082–11089. 39171663 10.1021/acs.nanolett.4c03254

[53] Mogera U, Guo H, Namkoong M, Rahman MS, Nguyen T, Tian L. Wearable plasmonic paper–based microfluidics for continuous sweat analysis. Sci Adv. 2022;8(12):eabn1736. 35319971 10.1126/sciadv.abn1736 PMC8942375

[54] Li Y, Guo Y, Chen H, Xiao X, Long F, Zhong H, Wang K, Guo Z, Zhuang Z, Liu Z. Flexible wearable plasmonic paper-based microfluidics with expandable channel and adjustable flow rate for portable surface-enhanced Raman scattering sweat sensing. ACS Photonics. 2024;11(2):613–625.

[55] Tseng C-C, Kung C-T, Chen R-F, Tsai M-H, Chao H-R, Wang Y-N, Fu LM. Recent advances in microfluidic paper-based assay devices for diagnosis of human diseases using saliva, tears and sweat samples. Sensors Actuators B Chem. 2021;342: Article 130078.

[56] Zhang X, Chen G, Yu Y, Sun L, Zhao Y. Bioinspired adhesive and antibacterial microneedles for versatile transdermal drug delivery. Research. 2020;2020: Article 3672120. 32490376 10.34133/2020/3672120 PMC7231261

[57] Huang X, Chen L, Sha T, Lin Y, Zeng R, Xu J, Chen S, Cai HH, Zhang J, Zhou H, et al. In situ tyrosinase monitoring by wearable microneedle patch toward clinical melanoma screening. ACS Nano. 2023;17(20):20073–20086. 37792448 10.1021/acsnano.3c05638

[58] Ju J, Hsieh C-M, Tian Y, Kang J, Chia R, Chang H, Bai Y, Xu C, Wang X, Liu Q. Surface enhanced Raman spectroscopy based biosensor with a microneedle array for minimally invasive in vivo glucose measurements. ACS Sens. 2020;5(6):1777–1785. 32426978 10.1021/acssensors.0c00444

[59] Mei R, Wang Y, Zhao X, Shi S, Wang X, Zhou N, Shen D, Kang Q, Chen L. Skin interstitial fluid-based SERS tags labeled microneedles for tracking of peritonitis progression and treatment effect. ACS Sens. 2023;8(1):372–380. 36638363 10.1021/acssensors.2c02409

[60] Xiao J, Zhang S, Liu Q, Xu T, Zhang X. Microfluidic-based plasmonic microneedle biosensor for uric acid ultrasensitive monitoring. Sensors Actuators B Chem. 2024;398: Article 134685.

[61] Koh EH, Lee W-C, Choi Y-J, Moon J-I, Jang J, Park S-G, Choo J, Kim DH, Jung HS. A wearable surface-enhanced Raman scattering sensor for label-free molecular detection. ACS Appl Mater Interfaces. 2021;13(2):3024–3032. 33404230 10.1021/acsami.0c18892

[62] Huang J-A, Mousavi MZ, Zhao Y, Hubarevich A, Omeis F, Giovannini G, Schütte M, Garoli D, de Angelis F. SERS discrimination of single DNA bases in single oligonucleotides by electro-plasmonic trapping. Nat Commun. 2019;10(1):5321. 31757965 10.1038/s41467-019-13242-x PMC6874578

[63] Plou J, García I, Charconnet M, Astobiza I, García-Astrain C, Matricardi C, Mihi A, Carracedo A, Liz-Marzán LM. Multiplex SERS detection of metabolic alterations in tumor extracellular media. Adv Funct Mater. 2020;30(17):1910335.

[64] Fan X, Zhao X, Tang X, Li G, Wei Y, Chen D, Kong F, Lan L, Wang J, Hao Q, et al. High-specificity SERS sensing with magnet-powered hierarchically structured micromotors. Opt Lett. 2024;49(24):7106–7109. 39671653 10.1364/OL.543066

[65] Sun X. Glucose detection through surface-enhanced Raman spectroscopy: A review. Anal Chim Acta. 2022;1206: Article 339226. 35473867 10.1016/j.aca.2021.339226

[66] Yadav S, Sadique MA, Ranjan P, Kumar N, Singhal A, Srivastava AK, Khan R. SERS based lateral flow immunoassay for point-of-care detection of SARS-CoV-2 in clinical samples. ACS Appl Bio Mater. 2021;4(4):2974–2995. 10.1021/acsabm.1c00102 35014387

[67] Chen Z, Wang W, Tian H, Yu W, Niu Y, Zheng X, Liu S, Wang L, Huang Y. Wearable intelligent sweat platform for SERS-AI diagnosis of gout. Lab Chip. 2024;24(7):1996–2004. 38373026 10.1039/d3lc01094e

[68] Sun D, Cao F, Wang H, Guan S, Su A, Xu W, Xu S. SERS hydrogel pellets for highly repeatable and reliable detections of significant small biomolecules in complex samples without pretreatment. Sensors Actuators B Chem. 2021;327: Article 128943.

[69] Guan P-C, Qi Q-J, Wang Y-Q, Lin J-S, Zhang Y-J, Li J-F. Development of a 3D hydrogel SERS chip for noninvasive, real-time pH and glucose monitoring in sweat. ACS Appl Mater Interfaces. 2024;16(36):48139–48146. 39197856 10.1021/acsami.4c10817

[70] Wu Y, Chen J-Y, He W-M. Surface-enhanced Raman spectroscopy biosensor based on silver nanoparticles@metal-organic frameworks with peroxidase-mimicking activities for ultrasensitive monitoring of blood cholesterol. Sensors Actuators B Chem. 2022;365: Article 131939.

[71] Lin J, Zhang D, Yu J, Pan T, Wu X, Chen T, Gao C, Chen C, Wang X, Wu A. Amorphous nitrogen-doped carbon nanocages with excellent SERS sensitivity and stability for accurate identification of tumor cells. Anal Chem. 2023;95(10):4671–4681. 36735867 10.1021/acs.analchem.2c05272

[72] Zhao X, Liu X, Chen D, Shi G, Li G, Tang X, Zhu X, Li M, Yao L, Wei Y, et al. Plasmonic trimers designed as SERS-active chemical traps for subtyping of lung tumors. Nat Commun. 2024;15(1):5855. 38997298 10.1038/s41467-024-50321-0 PMC11245553

[73] Lim WY, Goh C-H, Thevarajah TM, Goh BT, Khor SM. Using SERS-based microfluidic paper-based device (μPAD) for calibration-free quantitative measurement of AMI cardiac biomarkers. Biosens Bioelectron. 2020;147: Article 111792. 31678828 10.1016/j.bios.2019.111792

[74] Linh VTN, Lee M-Y, Mun J, Kim Y, Kim H, Han IW, Park SG, Choi S, Kim DH, Rho J, et al. 3D plasmonic coral nanoarchitecture paper for label-free human urine sensing and deep learning-assisted cancer screening. Biosens Bioelectron. 2023;224: Article 115076. 36641876 10.1016/j.bios.2023.115076

[75] Meng X, Yu J, Shi W, Qiu L, Qiu K, Li A, Liu Z, Wang Y, Wu J, Lin J, et al. SERS detection of trace carcinogenic aromatic amines based on amorphous MoO3 monolayers. Angew Chem Int Ed Engl. 2024;136(33): Article e202407597. 10.1002/anie.202407597 38818663

[76] Masterson AN, Hati S, Ren G, Liyanage T, Manicke NE, Goodpaster JV, Sardar R. Enhancing nonfouling and sensitivity of surface-enhanced Raman scattering substrates for potent drug analysis in blood plasma via fabrication of a flexible plasmonic patch. Anal Chem. 2021;93(4):2578–2588. 33432809 10.1021/acs.analchem.0c04643

[77] Lin C, Liu Z, Fang F, Zhao S, Li Y, Xu M, Peng Y, Chen H, Yuan F, Zhang W, et al. Next-generation rapid and ultrasensitive lateral flow immunoassay for detection of SARS-CoV-2 variants. ACS Sens. 2023;8(10):3733–3743. 37675933 10.1021/acssensors.3c01019

[78] Chen D, Zhang L, Ning P, Yuan H, Zhang Y, Zhang M, Fu T, He X. In-situ growth of gold nanoparticles on electrospun flexible multilayered PVDF nanofibers for SERS sensing of molecules and bacteria. Nano Res. 2021;14:4885–4893.

[79] Li A, Yao C, Xia J, Wang H, Cheng Q, Penty R, Fainman Y, Pan S. Advances in cost-effective integrated spectrometers. Light Sci Appl. 2022;11:174. 35672298 10.1038/s41377-022-00853-1 PMC9174208

[80] Crocombe R, Kammrath B, Leary P. Portable Raman spectrometers: How small can they get? Spectrosc Suppl. 2023;38(S6):32–40.

[81] Scheeline A. How to design a spectrometer. Appl Spectrosc. 2017;71(10):2237–2252. 28644044 10.1177/0003702817720468

[82] Edwards P, Zhang C, Zhang B, Hong X, Nagarajan VK, Yu B, Liu Z. Smartphone based optical spectrometer for diffusive reflectance spectroscopic measurement of hemoglobin. Sci Rep. 2017;7(1):12224. 28939898 10.1038/s41598-017-12482-5 PMC5610341

[83] Gao B, Shi Z, Boyd RW. Design of flat-band superprism structures for on-chip spectroscopy. Opt Express. 2015;23(5):6491–6496. 25836867 10.1364/OE.23.006491

[84] Neumann N, Ebermann M, Kurth S, Hiller K. Tunable infrared detector with integrated micromachined Fabry-Perot filter. J Micro/Nanolith MEMS MOEMS. 2008;7(2):021004.

[85] Tittl A, Leitis A, Liu M, Yesilkoy F, Choi D-Y, Neshev DN, Kivshar YS, Altug H. Imaging-based molecular barcoding with pixelated dielectric metasurfaces. Science. 2018;360(6393):1105–1109. 29880685 10.1126/science.aas9768

[86] Emadi A, Wu H, de Graaf G, Wolffenbuttel R. Design and implementation of a sub-nm resolution microspectrometer based on a linear-variable optical filter. Opt Express. 2011;20(1):489–507. 10.1364/OE.20.000489 22274371

[87] Hirschfeld T. Fellgett’s advantage in UV-VIS multiplex spectroscopy. Appl Spectrosc. 1976;30(1):68–69.

[88] Kita DM, Miranda B, Favela D, Bono D, Michon J, Lin H, Gu T, Hu J. High-performance and scalable on-chip digital Fourier transform spectroscopy. Nat Commun. 2018;9(1):4405. 30353014 10.1038/s41467-018-06773-2 PMC6199339

[89] Waldron A, Allen A, Colón A, Carter JC, Angel SM. A monolithic spatial heterodyne Raman spectrometer: Initial tests. Appl Spectrosc. 2021;75(1):57–69. 32495633 10.1177/0003702820936643

[90] Yao C, Xu K, Zhang W, Chen M, Cheng Q, Penty R. Integrated reconstructive spectrometer with programmable photonic circuits. Nat Commun. 2023;14:6376. 37821463 10.1038/s41467-023-42197-3 PMC10567699

[91] Grotevent MJ, Yakunin S, Bachmann D, Romero C, Vázquez de Aldana JR, Madi M, Calame M, Kovalenko MV, Shorubalko I. Integrated photodetectors for compact Fourier-transform waveguide spectrometers. Nat Photonics. 2023;17:59–64. 36628352 10.1038/s41566-022-01088-7 PMC9822831

[92] Ilchenko O, Pilhun Y, Kutsyk A, Slobodianiuk D, Goksel Y, Dumont E, Vaut L, Mazzoni C, Morelli L, Boisen S, et al. Optics miniaturization strategy for demanding Raman spectroscopy applications. Nat Commun. 2024;15(1):3049. 38589380 10.1038/s41467-024-47044-7 PMC11001912

[93] Acosta JN, Falcone GJ, Rajpurkar P, Topol EJ. Multimodal biomedical AI. Nat Med. 2022;28(9):1773–1784. 36109635 10.1038/s41591-022-01981-2

[94] Zhang Y, Chang K, Ogunlade B, Herndon L, Tadesse LF, Kirane AR, Dionne JA. From genotype to phenotype: Raman spectroscopy and machine learning for label-free single-cell analysis. ACS Nano. 2024;18(28):18101–18117. 38950145 10.1021/acsnano.4c04282

[95] Ying X, Leng S-Y, Ma H-F, Nie Q, Lai Y-C, Lin W. Continuity scaling: A rigorous framework for detecting and quantifying causality accurately. Research. 2022;2022: Article 9870149. 35600089 10.34133/2022/9870149 PMC9101326

[96] Zhang C, Xu L, Miao X, Zhang D, Xie Y, Hu Y, Zhang Z, Wang X, Wu X, Liu Z, et al. Machine learning assisted dual-modal SERS detection for circulating tumor cells. Biosens Bioelectron. 2025;268: Article 116897. 39488132 10.1016/j.bios.2024.116897

[97] Xie Y, Xu L, Zhang J, Zhang C, Hu Y, Zhang Z, Chen G, Qi S, Xu X, Wang J, et al. Precise diagnosis of tumor cells and hemocytes using ultrasensitive, stable, selective cuprous oxide composite SERS bioprobes assisted with high-efficiency separation microfluidic chips. Mater Horiz. 2024;11(22):5752–5767. 39264270 10.1039/d4mh00791c

[98] Cui F, Yue Y, Zhang Y, Zhang Z, Zhou HS. Advancing biosensors with machine learning. ACS Sens. 2020;5(11):3346–3364. 33185417 10.1021/acssensors.0c01424

[99] Xie Y, Chen C, Zhang C, Xu L, Li Z, Ren W, Xu X, Ren Y, Lin J, Wu A. Synergistic enhancement of ultrahigh SERS activity via Cu2O@Ag Core-Shell structure for accurate label-free identification of breast tumor subtypes. Nano Today. 2024;54: Article 102140.

[100] Erzina M, Trelin A, Guselnikova O, Dvorankova B, Strnadova K, Perminova A, Ulbrich P, Mares D, Jerabek V, Elashnikov R, et al. Precise cancer detection via the combination of functionalized SERS surfaces and convolutional neural network with independent inputs. Sensors Actuators B Chem. 2020;308: Article 127660.

[101] Yang Y, Li H, Jones L, Murray J, Haverstick J, Naikare HK, Mosley YYC, Tripp RA, Ai B, Zhao Y. Rapid detection of SARS-CoV-2 RNA in human nasopharyngeal specimens using surface-enhanced Raman spectroscopy and deep learning algorithms. ACS Sens. 2022;8(1):297–307. 36563081 10.1021/acssensors.2c02194

[102] Horta-Velázquez A, Arce F, Rodríguez-Sevilla E, Morales-Narváez E. Toward smart diagnostics via artificial intelligence-assisted surface-enhanced Raman spectroscopy. Trends Anal Chem. 2023;169: Article 117378.

[103] Shen J, Li M, Li Z, Zhang Z, Zhang X. Single convolutional neural network model for multiple preprocessing of Raman spectra. Vib Spectrosc. 2022;121: Article 103391.

[104] Wahl J, Sjödahl M, Ramser K. Single-step preprocessing of Raman spectra using convolutional neural networks. Appl Spectrosc. 2020;74(4):427–438. 31961223 10.1177/0003702819888949

[105] Xu J, Yi X, Jin G, Peng D, Fan G, Xu X, Chen X, Yin H, Cooper JM, Huang WE. High-speed diagnosis of bacterial pathogens at the single cell level by Raman microspectroscopy with machine learning filters and denoising autoencoders. ACS Chem Biol. 2022;17(2):376–385. 35026119 10.1021/acschembio.1c00834

[106] Kazemzadeh M, Martinez-Calderon M, Xu W, Chamley LW, Hisey CL, Broderick NGR. Cascaded deep convolutional neural networks as improved methods of preprocessing Raman spectroscopy data. Anal Chem. 2022;94(37):12907–12918. 36067379 10.1021/acs.analchem.2c03082

[107] Lin Y, Lin J, Zheng M, Gong W, Li H, Shu Z, du W, Gao S, Yu Y. Quantitative and direct serum albumin detection by label-free SERS using tunable hydroxyapatite nanostructure for prostate cancer detection. Anal Chim Acta. 2022;1221: Article 340101. 35934347 10.1016/j.aca.2022.340101

[108] Han S, Chen C, Chen C, Wu L, Wu X, Lu C, Zhang X, Chao P, Lv X, Jia Z, et al. Coupling annealed silver nanoparticles with a porous silicon Bragg mirror SERS substrate and machine learning for rapid non-invasive disease diagnosis. Anal Chim Acta. 2023;1254: Article 341116. 37005026 10.1016/j.aca.2023.341116

[109] Xie Y, Su X, Wen Y, Zheng C, Li M. Artificial intelligent label-free SERS profiling of serum exosomes for breast cancer diagnosis and postoperative assessment. Nano Lett. 2022;22(19):7910–7918. 36149810 10.1021/acs.nanolett.2c02928

[110] Premachandran S, Haldavnekar R, Das S, Venkatakrishnan K, Tan B. DEEP surveillance of brain cancer using self-functionalized 3D nanoprobes for noninvasive liquid biopsy. ACS Nano. 2022;16(11):17948–17964. 36112671 10.1021/acsnano.2c04187

[111] Phyo JB, Woo A, Yu HJ, Lim K, Cho BH, Jung HS, Lee MY. Label-free SERS analysis of urine using a 3D-stacked AgNW-glass fiber filter sensor for the diagnosis of pancreatic cancer and prostate cancer. Anal Chem. 2021;93(8):3778–3785. 33576598 10.1021/acs.analchem.0c04200

[112] Shin H, Choi BH, Shim O, Kim J, Park Y, Cho SK, Kim HK, Choi Y. Single test-based diagnosis of multiple cancer types using exosome-SERS-AI for early stage cancers. Nat Commun. 2023;14(1):1644. 36964142 10.1038/s41467-023-37403-1 PMC10039041

[113] Xie X, Yu W, Wang L, Yang J, Tu X, Liu X, Liu S, Zhou H, Chi R, Huang Y. SERS-based AI diagnosis of lung and gastric cancer via exhaled breath. Spectrochim Acta A Mol Biomol Spectrosc. 2024;314: Article 124181. 38527410 10.1016/j.saa.2024.124181

[114] Wu L, Teixeira A, Garrido-Maestu A, Muinelo-Romay L, Lima L, Santos LL, Prado M, Diéguez L. Profiling DNA mutation patterns by SERS fingerprinting for supervised cancer classification. Biosens Bioelectron. 2020;165: Article 112392. 32729513 10.1016/j.bios.2020.112392

[115] Li G, Hao Q, Li M, Zhao X, Song W, Fan X, Qiu T. Quantitative SERS analysis by employing Janus nanoparticles with internal standards. Adv Mater Interfaces. 2023;10(7):2202127.

[116] Bi X, Czajkowsky DM, Shao Z, Ye J. Digital colloid-enhanced Raman spectroscopy by single-molecule counting. Nature. 2024;628:771–775. 38632399 10.1038/s41586-024-07218-1

